# How Temperature Affects the Selectivity of the Electrochemical CO_2_ Reduction on Copper

**DOI:** 10.1021/acscatal.3c00706

**Published:** 2023-06-01

**Authors:** Rafaël E. Vos, Kees E. Kolmeijer, Thimo S. Jacobs, Ward van der Stam, Bert M. Weckhuysen, Marc T. M. Koper

**Affiliations:** † Leiden Institute of Chemistry, Leiden University, P.O.Box 9502, 2300 RA Leiden, The Netherlands; ‡ Inorganic Chemistry and Catalysis group, Debye Institute for Nanomaterials Science and Institute for Sustainable and Circular Chemistry, Utrecht University, Universiteitsweg 99, 3584 CG Utrecht, The Netherlands

**Keywords:** CO_2_ reduction, temperature, copper, selectivity, CO coverage, Raman spectroscopy, surface reconstruction

## Abstract

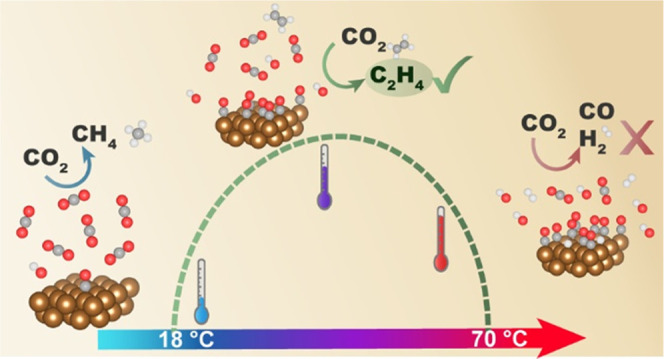

Copper is a unique catalyst for the electrochemical CO_2_ reduction reaction (CO2RR) as it can produce multi-carbon products, such as ethylene and propanol. As practical electrolyzers will likely operate at elevated temperatures, the effect of reaction temperature on the product distribution and activity of CO2RR on copper is important to elucidate. In this study, we have performed electrolysis experiments at different reaction temperatures and potentials. We show that there are two distinct temperature regimes. From 18 up to ∼48 °C, C2+ products are produced with higher Faradaic efficiency, while methane and formic acid selectivity decreases and hydrogen selectivity stays approximately constant. From 48 to 70 °C, it was found that HER dominates and the activity of CO2RR decreases. Moreover, the CO2RR products produced in this higher temperature range are mainly the C1 products, namely, CO and HCOOH. We argue that CO surface coverage, local pH, and kinetics play an important role in the lower-temperature regime, while the second regime appears most likely to be related to structural changes in the copper surface.

## 1 Introduction

Electrochemical CO_2_ reduction (CO2RR) is an interesting process as it exhibits the unique ability to use CO_2_ as a resource to produce renewable feedstocks for the chemical industry or so-called solar fuels, which can be used within a future renewable energy system.^1,2^ Many different catalysts can be used, of which copper is remarkable as it is practically the only catalyst able to make multi-carbon products such as ethylene, ethanol, and propanol. Although other metals such as Ag have shown to be able to produce these products,^3^ copper is the only monometallic metal that can produce them at significant Faradaic efficiencies (FEs).^4−6^ However, copper is not very selective and produces a complex mixture of both gaseous and liquid reaction products.^7^ To be able to employ CO2RR on an industrial level, an FE toward a single product of over 90% is needed.^8,9^ Therefore, it is crucial that both the selectivity and activity of the catalysts are further improved, for example, by tuning the reaction conditions.^8−11^

Reaction temperature is an important but often neglected parameter in the field of electrochemistry in general and for CO_2_ reduction in particular. Often a perceived benefit of electrocatalysis over thermal catalysis is that the former can be performed at room temperature and ambient pressures. However, in practice,^12^ electrolyzers will always operate at elevated temperatures, for example, due to thermal losses^13−15^ and/or hot feedstocks. In a previous paper,^16^ we have investigated the effect of temperature on a simple CO2RR system using gold as a catalyst. We found that on gold it is beneficial to perform CO2RR at increased temperature as both the selectivity and the activity toward CO increase. However, mass transport becomes more important at elevated temperatures and at a certain point, CO_2_ availability becomes the limiting factor. From 55 °C onward, we observed a plateau in the activity of CO_2_ reduction, even under highly efficient mass transport conditions.

Several other studies have shown the effect of temperature on CO2RR on other simple electrode systems, such as Ag^17^ and Sn.^18−20^ However, for copper, still not all selectivity trends with temperature have been well identified. Ahn et al.^21^ have shown that the selectivity toward methane decreases with reaction temperature, whereas hydrogen dominates at higher temperatures at −1.6 V vs Ag/AgCl. They also observed an optimum in ethylene selectivity around 22 °C. The trends in CO and HCOOH selectivity with temperature are unclear, although in a recent study, a decreasing trend with temperature was observed for the HCOOH selectivity on a copper foam.^22^ Hori^23^ showed similar results using galvanostatic electrolysis, as he found that hydrogen selectivity is increasing and methane selectivity is decreasing with temperature. However, Hori observed different trends for the selectivity of ethylene and CO compared to Ahn et al. as they did not observe an optimum in ethylene production, whereas CO selectivity seemed to increase with temperature. Although Zong et al.^24^ mainly focused on obtaining apparent activation energies of the different products of CO2RR on Cu at different potentials, they did show that the Faradaic efficiency for hydrogen increases with temperature, while methane selectivity decreases, in agreement with the works of Ahn and Hori. However, again the trends in CO and C_2_H_4_ selectivity with temperature are less well defined.

Even though there exists some literature about the temperature effect on CO2RR over copper, there remains a significant gap in our understanding. All studies discussed above were limited in their temperature range to a maximum of 45 °C, while industrial electrolyzers will not be limited to these temperatures.^12^ Moreover, optima in selectivity for CO2RR at other electrode materials were found at higher temperatures.^16,18^ Additionally, although clear trends can be observed for methane and hydrogen selectivity, for the other products, the exact trends with temperature remain unresolved. Lastly, previous work has not provided detailed explanations of the trends observed; most ascribe the trends simply to the balance of kinetics and CO_2_ solubility, without testing alternatives.

In this study, we investigate how reaction temperature affects the electrochemical CO_2_ reduction on copper in the temperature range from 18 to 70 °C. We show that different products display different trends with temperature. CO selectivity remains relatively stable, while the CH_4_ and HCOOH selectivities decrease with temperature. The selectivity toward C2+ products shows an optimum around 48 °C, whereas hydrogen formation dominates at higher temperatures. We identify two regimes in this temperature range, the first up to ∼48 °C, in which CO2RR activity increases, and the second above 48 °C, in which CO_2_ reactivity declines. The observations in the first regime can be explained by an increase in the CO coverage on the surface as evidenced by in situ Raman spectroscopy, in combination with increased local pH and faster kinetics with temperature. The second regime seems not to be related to any limited CO_2_ concentration but rather due to changes in the copper surface as shown with lead underpotential deposition and double layer capacitance measurements, although a too high local pH could also be a factor.

## 2 Experimental Section

### 2.1 Chemicals

The electrolytes were prepared from KHCO_3_ (99.95%, Sigma-Aldrich), KOH (99.99%, Sigma-Aldrich), K_2_SO_4_ (99.999% Suprapur, Sigma-Aldrich), NaClO_4_ (Emsure, Sigma-Aldrich), HClO_4_ (60%, Emsure, Sigma-Aldrich), NaCl (99.99% Suprapur, Merck), Pb(ClO_4_)_2_ (99.995%, Sigma-Aldrich), and Milli-Q water (≥18.2 MΩ cm, TOC < 5 ppb). H_2_SO_4_ (95–98%, Sigma-Aldrich), H_2_O_2_ (35%, Merck), and KMnO_4_ (99%, Sigma-Aldrich) were used to clean the cells. The KHCO_3_ and KOH + K_2_SO_4_ electrolytes were stored with Chelex (100 sodium form, Sigma-Aldrich) to clean the electrolyte from any metal impurities.^25^ The KOH + K_2_SO_4_ electrolyte was stored in a plastic container to prevent contamination by leaching of metals from glass. Ar (5.0 purity, Linde), He (5.0 purity, Linde), CO (4.7 purity, Linde), and CO_2_ (4.5 purity, Linde) were used for purging the electrolytes.

### 2.2 General Electrochemical Methods

The experiments were performed in a homemade PEEK H-cell or a borosilicate glass cell, which were cleaned prior to experiments by storing in permanganate solution overnight (0.5 M H_2_SO_4_, 1 g/L KMnO_4_). Before use, the cell was rinsed, submerged in diluted piranha acid solution to remove any traces of MnO_4_ and MnO_2_, rinsed again, and boiled three times with Milli-Q water. The polycrystalline Cu working electrode (99.99%, Mateck) was first mechanically polished with a diamond polishing suspension of decreasing particle size (3.0, 1.0, and 0.25 μm, Buehler) on micropolishing cloths (8 inch). After polishing, the electrode was successively sonicated in ethanol and Milli-Q water for 3 min to remove any impurities and dried with pressurized air. The Cu disk was then electrochemically polished in a solution of H_3_PO_4_ (85%, Suprapure, Merck) by applying +3 V versus a graphite counter electrode for 20 s and subsequently rinsed with Milli-Q water. All of the electrochemical measurements were carried out using an IviumStat potentiostat (Ivium Technologies).

### 2.3 Electrolysis Experiments

The electrolysis experiments were performed in the homemade PEEK H-cell containing 7.5 mL of 0.1 M KHCO_3_ electrolyte in each compartment; see Figures S1 and S2 for details of the cell employed. For the CO reduction experiments 0.1 M KOH + 0.2 M K_2_SO_4_ was used as the electrolyte instead of KHCO_3_. The PEEK H-cell was embedded in a jacket, which was connected to the water bath (Ecoline e100, Lauda) to control the temperature in the cell. The temperature of the electrolyte in the cell was calibrated to the temperature of the jacket using a thermocouple in the cell before the actual CO2RR experiments. Low partial current densities have been used to prevent heating of the electrode.^14,15^ Experiments were performed in a three-electrode configuration with the reference electrode in the same compartment as the working electrode, which had a geometric surface area of 0.785 cm^2^. The reference electrode was a commercial reversible hydrogen electrode (RHE) (mini Hydroflex, Gaskatel). The counter electrode was a dimensionally stable anode (DSA, Magneto) and was separated from the working electrode by an anion exchange membrane (AMVN Selemion, AGC). Before electrolysis, CO_2_ was purged through the electrolyte for 15 min while controlling the potential at −0.1 V vs RHE to saturate the electrolyte and heat the electrolyte to the proper temperature. The flow of CO_2_ or CO (and He for the partial pressure experiments) was controlled using a mass flow controller (SLA5850, Brooks Instrument). Next, the Ohmic resistance was determined by electrochemical impedance spectroscopy (EIS) at −0.1 V vs RHE and 85% Ohmic drop compensation was performed for all chronoamperometry measurements. Chronoamperometry was performed for 60 min, while CO_2_ was constantly purged through a PEEK-frit (0.2 um pore size, IDEX) at 40 mL/min to increase mass transport in the cell.^13,26^ At 5, 19, 32, 46, and 60 min, a gas sample was analyzed online using a Shimadzu 2014 gas chromatograph with two detectors (one TCD with a Shincarbon column and one FID with a RTX-1 column). At the end of the electrolysis, a liquid sample was taken and analyzed using high-performance liquid chromatography (HPLC, Shimadzu) with an Aminex HPX-87H column (Bio-rad). The 5 gas samples were averaged and combined with the HPLC data to obtain the selectivities and activities of the different products for a single experiment. The values reported are averages of at least three repetitions with twice the standard deviation as the reported error bars.^27−30^ The sum of the FEs for the major products (hydrogen, CO, methane, ethylene, ethanol, formic acid, and propanol) for every individual measurements is between 85 and 102%.

### 2.4 Efficiency Calculations

Because the Faradaic efficiencies (FEs) become dominated by hydrogen at higher temperatures, we have defined a carbon efficiency (CE) to obtain better insights into how CO2RR activity itself changes at higher temperature. The CE is defined equivalently to the FE
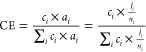
1where *c*_*i*_ is the number of carbon atoms in product *i*, *a*_*i*_ is the production rate of *i* in mol/min, *j*_*i*_ is the corresponding partial current density, and *n*_*i*_ is the number of electrons transferred during CO2RR for product *i*.

### 2.5 Partial Pressure Experiments

With the use of flow controllers, the partial pressure of CO_2_ can be changed by mixing the inlet flow with He gas. He was chosen instead of Ar as He is the carrier gas used in the GC, so it would not lead to broad extra peaks in the chromatogram. Controlling the partial pressure allows us to change the CO_2_ concentration in the bulk electrolyte independently of temperature. We estimate the CO_2_ concentration at different temperatures by using Henry’s law in combination with an empirical equation to estimate Henry’s constant.^31^
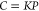
2
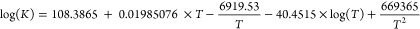
3where *C* is the concentration, *K* is Henry’s constant, *P* is the partial pressure, and *T* is the temperature.

### 2.6 Reversibility Experiment

To assess whether observed changes in selectivity with temperature are reversible, “reversibility experiments” were carried out. For the reversibility experiments, the measurement was started at −1.1 V vs RHE at 70 °C (or 48 °C for the control experiments) in a similar way to the other electrolysis experiment. However, after 20 min, the water bath was changed with water at 35 °C to cool the cell down for 8 min. Then, the temperature was increased to 48 °C and was maintained for the rest of the experiment. During this cooling and reheating of the electrolyte for 15 min in total, the potential was maintained at −0.1 V vs RHE. Then, the resistance was measured again and chronoamperometry was performed at −1.1 V vs RHE for another 32 min.

### 2.7 Surface Area Determination

The electrochemical surface area was determined from the double layer capacitance. These were measured following the protocol of Morales et al.^32^ The potential was scanned in a broad potential range, namely, −0.2 to 0.3 V vs RHE at sufficiently high scan rates (200–1400 mV/s). The capacitance was determined from the current width between the anodic and cathodic scan at 0.0 V vs RHE plotted against the scan rate. The slope of this graph gives the double layer capacitance. These measurements were performed before CO2RR and after 15 min and 30 min of CO2RR at the different temperatures.

### 2.8 Raman Experiments

In situ surface-enhanced Raman Spectroscopy (SERS) was performed using an inverted confocal Raman microscope (LabRam HR, Horiba Jobin Yvon with a 50× objective and grating of 1800 lines/mm). A He/Ne laser (633 nm) was used as an excitation source, which has an intensity of 8.5 mW at the sample without density filter. The acquisition time of all of the Raman spectra was 10 s. An edge filter at 633 nm was used to filter the backscattered light, which was subsequently directed to the spectrograph and to the CCD detector; further details of the setup can be found in refs (33) and (34). The experiments were performed in a homemade jacketed three-electrode cell made of borosilicate glass, with a quartz window at the bottom. The jacket was connected to the water bath to heat the electrolyte. A platinum mesh was used as the counter electrode, a Hydroflex RHE as the reference electrode, and a roughened copper disk as the working electrode. The copper was first polished as described above and consequently roughened by applying −1.8 V vs a copper counter electrode for 25 s in 0.1 M H_2_SO_4_ + 0.1 M CuSO_4_. The same roughened copper electrode was used at all measured temperatures (i.e., 20, 30, 40, 50, and 60 °C), and at each temperature, 10 different, randomly picked spots on the electrode were measured. The obtained spectra were baseline-corrected using the SNIP algorithm for background elimination.^35^ After the background correction, a Gaussian fit was performed on the individual spectra to calculate the area of the 280 and 360 cm^–1^ peaks. The Raman shift window between 250 and 400 cm^–1^ was used for the fits, with a set boundary between the two peaks at 310 cm^–1^. An example of the background correction and the fit can be found in Figure S11. The ratio of the 360 and 280 cm^–1^ peak areas was used to determine the CO coverage, following the methodology used by Zhan et al.^36^ The ratio of the different spots was averaged, and only spectra where the peak intensity of the 360 cm^–1^ peak is above 500 counts were taken into account. The coverage was determined at −0.7 and −0.95 V vs RHE. At −1.1 V, too many bubbles were produced to obtain sufficient spectra with a decent signal-to-noise ratio, so we decided not to take this potential into account.

### 2.9 Lead (Pb) Underpotential Deposition Experiments

Lead underpotential deposition (UPD) experiments were performed to examine the copper surface for changes after electrolysis. For the Pb UPD experiments, the procedure of Sebastián-Pascual et al.^37^ was followed. First, electrolysis was performed for 20 min at the desired temperature in 0.1 M KHCO_3_. Then, the electrode was rinsed, dried with air, and transferred to an Ar-purged glass cell containing a 0.1 M NaClO_4_ + 1 mM NaCl + 2 mM PbClO_4_ pH 3 electrolyte. A homemade RHE was used as the reference electrode and a gold wire as the counter electrode. The working electrode was introduced into the electrolyte while holding the potential at 0.3 V vs RHE, and subsequently a cyclic voltammetry (CV) measurement was performed from 0.3 to 0.0 V vs RHE at 5 mV/s, of which the second scan was used.

### 2.10 Characterization of Morphology and Chemical Composition

To examine the copper surface further and to inspect the surface for any deposits, scanning electron microscopy (SEM) combined with energy-dispersive X-ray spectroscopy (EDX) was performed. Micrographs of Cu after CO2RR at different temperatures (i.e., 25, 48 and 70 °C) were obtained in an Apreo scanning electron microscope (SEM, Thermo Fisher Scientific) with an acceleration voltage of 15 kV and an electron beam current of 0.4 nA. The chemical composition of the electrode was investigated by EDX using an Oxford Instruments X-MaxN 150 Silicon Drift detector coupled to the Apreo SEM. EDX data was processed with Pathfinder X-ray Microanalysis software v1.3. The quantification of chemical elements was performed in automatic mode and the chemical composition of the electrodes was determined by averaging the chemical compositions of at least 5 different sections of the electrode surface.

## 3 Results and Discussion

### 3.1 Trends in Selectivity with Temperature

**Figure 1:**
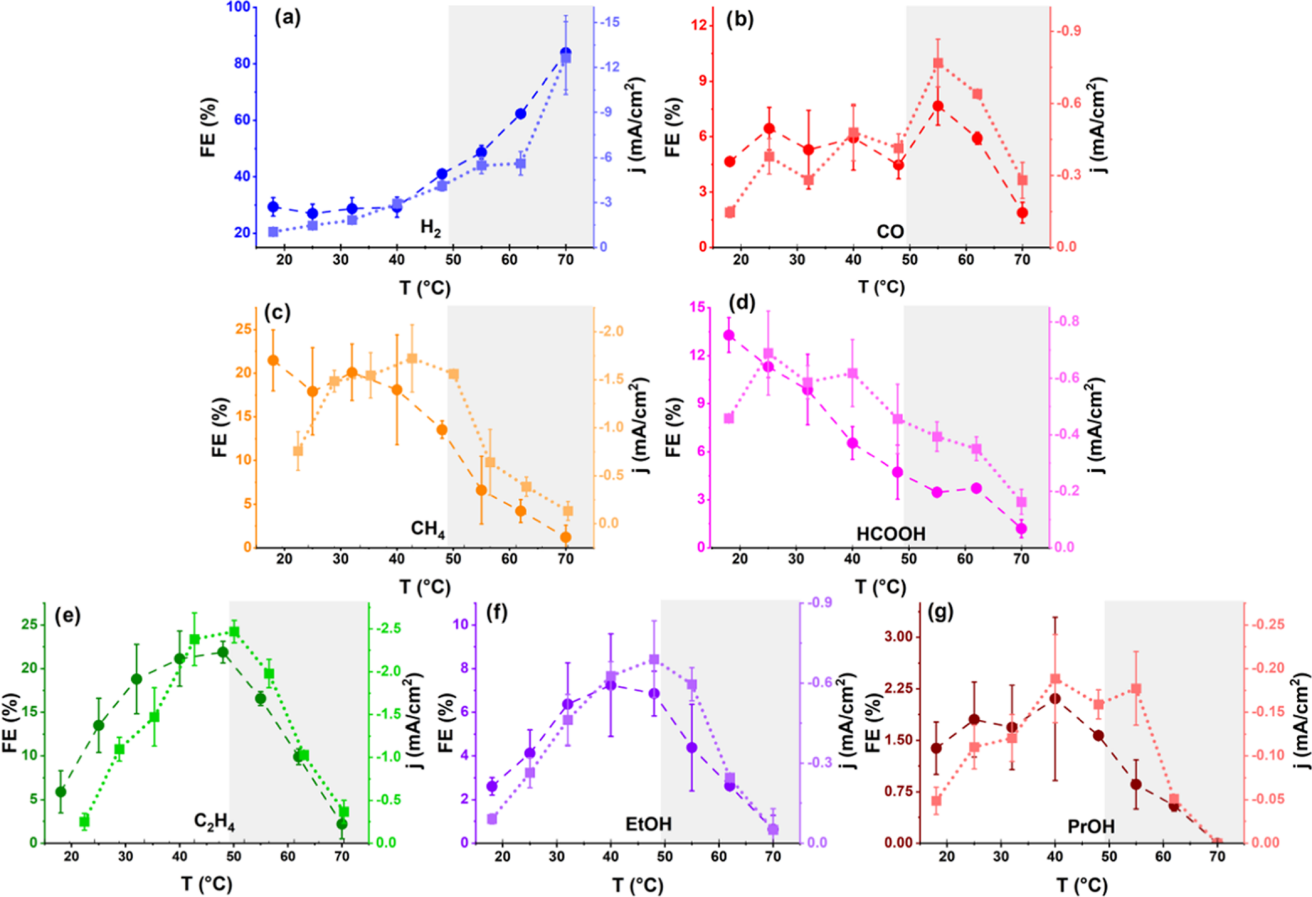
Faradaic efficiency (in dark circles) and partial current density (in light squares) during CO2RR at different reaction temperatures in 0.1 M KHCO_3_ at −1.1 V vs RHE for (a) hydrogen, (b) CO, (c) methane, (d) formic acid, (e) ethylene, (f) ethanol, and (g) 1-propanol. The error bars are determined from at least 3 separate experiments. The gray background indicates the second regime, and the dotted lines are a guide to the eye.

**Figure 2:**
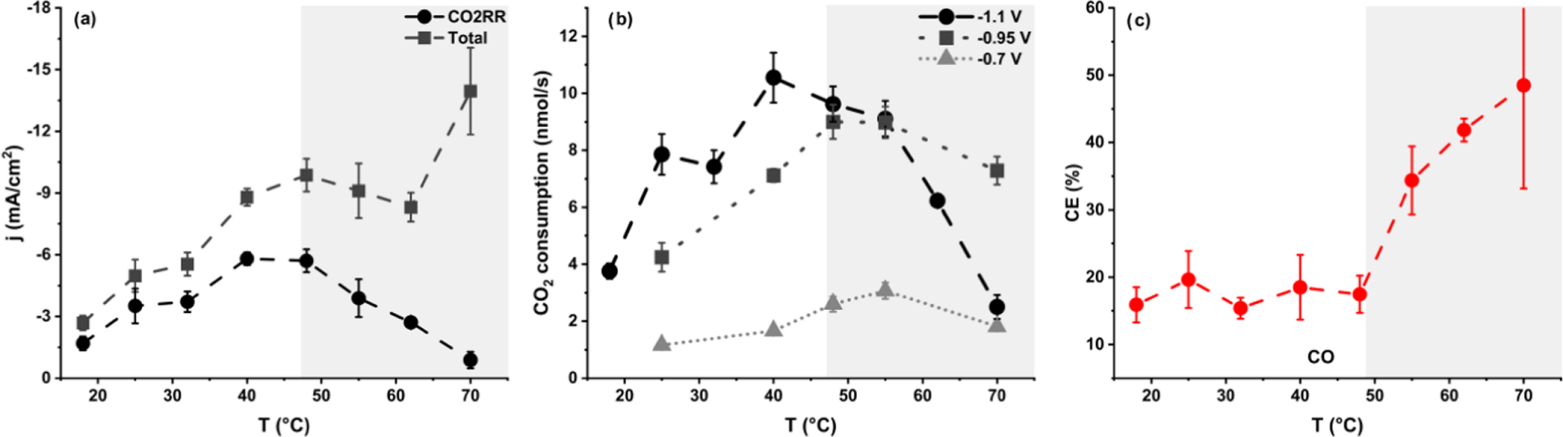
(a) Total current density and current density toward CO2RR at −1.1 V vs RHE. (b) Total consumption of CO_2_ during CO2RR at different reaction temperatures in 0.1 M KHCO_3_ at −1.1, −0.9, and −0.7 V vs RHE. (c) Carbon efficiency toward CO at −1.1 V vs RHE, CEs for other products can be found in Figure S3. The error bars are determined from at least 3 separate experiments. The gray background indicates the second regime, and the dotted lines are a guide to the eye.

Figure 1 shows the effect of reaction temperature on the product distribution of CO2RR on copper. This figure shows the Faradaic efficiencies (FEs) toward the most important products between 18 and 70 °C at −1.1 V vs RHE in dark circles. Several trends can be observed, which can be divided into two regimes. The first regime transitions into the second regime at around 48 °C. This exact transition temperature can be debated, but 48 °C has been chosen as ethylene shows an optimum in FE and carbon efficiency (CE) here, and the CE of CO starts to increase from this temperature onward. In the first regime, the hydrogen evolution reaction (HER) selectivity remains constant, while in the second regime, it increases significantly with temperature up to 84% FE at 70 °C. Of the CO_2_ reduction products, methane and formic acid show a strong decreasing trend with temperature in both regimes. The selectivity toward CO stays reasonably stable, except for the highest temperature at 70 °C. The C2+ products increase in the first regime, while they decrease in the second regime resulting in an optimum in FE around 40–48 °C. Figure 1 shows the corresponding activity trends of all of these products in the same temperature range in light squares. Initially, the activity toward all products increases with temperature, although the increase for the C2+ products is more significant than for the other carbon products. The activity towards the C2+ products shows an optimum at ∼40–48 °C, similar to the FE trend. The activity toward methane and formic acid clearly decreases from 40 °C onward, and CO shows an optimum around 55 °C. Liquid products, especially ethanol, might evaporate at elevated temperatures. However, we do not think that this significantly affects the results as we have not observed ethanol in the GC, and ethanol follows a similar trend as ethylene. The HER rate increases steadily with temperature and shows a large increase at around 70 °C, for which temperature there is also a sudden increase in overall current as can be seen in Figure 2a. This figure shows that the total current density increases with increasing temperature, although the current density decreases slightly from 48 to 62 °C due to the fast decrease in CO_2_ reduction activity, after which HER takes over and the total current density increases rapidly at higher temperatures.

Figure 2a,b shows that the total activity for CO_2_ reduction increases in the first regime and decreases in the second at −1.1 V vs RHE, regardless if we look at CO_2_ consumption or current density for CO2RR. The CO_2_ consumption has been back calculated from the partial current densities of the CO2RR products. To clearly identify the temperature effect on the CO2RR pathways, we define a carbon efficiency (CE), which is essentially the FE but excluding H_2_. The optimum found in CO_2_ consumption around 40–48 °C is also observed in CE for the C2+ products, as can be seen in Figure S3. The trends in CE are mostly similar to the trends in the FEs, although the two regimes are more pronounced in the CE graphs. Methane shows similar trends in FE and CE, although in the CE, a rapid decrease is visible when switching between regimes. On the other hand, CO and HCOOH show significantly different trends in the CE compared to the FE because the FE for H_2_ changes strongly with temperature. The CE toward CO (Figure 2c) remains stable in the first regime but increases significantly at high temperatures in the second regime. Formic acid shows decreasing CE in the first regime (just as for the FE), but in the second regime, it increases slightly. Therefore, at the higher temperatures, CO2RR mostly (>70% at 70 °C) produces the most simple C1 products, namely, HCOOH and CO.

Experiments at different potentials (−0.95 and −0.7 V) were performed to assess if these trends hold at other potentials as well. Figure 2b shows that the optimum in CO_2_ consumption is most pronounced at the highest overpotential. Figures S2 and S4 show the FE and CE at −0.95 V vs RHE, which was chosen as it is near the onset of C2+ formation. This results in the same products as at −1.1 V but generated at lower currents so that probably there is lower local pH and there are less pronounced mass transport limitations. Most of the trends are quite similar to −1.1 V vs RHE, although the FEs for the multi-carbon products are much lower than at −1.1 V vs RHE. There is an optimum in both CE and FE for C2+ products at 48 °C, whereas the hydrogen selectivity increases in the second regime. Main differences are that the CE toward HCOOH does not increase at high temperatures, instead, mainly CO is the favored carbon product. Moreover, the FE toward CO decreases with temperature, although the CE increases in the second regime similarly to the situation at −1.1 V. The decrease of FE for CO might be linked to the lack of CH_4_ formation. As there is already hardly any methane at 25 °C, its selectivity cannot decrease with temperature. However, ethylene, ethanol, and hydrogen still show an increased FE with temperature, so that the FE for some other product(s), i.e., CO and HCOOH, should be decreasing.

Figures S6 and S7 show the FE and CE at −0.7 V vs RHE, which was chosen as only HCOOH, CO, and H_2_ are produced. At this potential, similar trends in FE are observed; HCOOH decreases and CO remains reasonably stable except at 70 °C, at which temperature it drops rapidly. Hydrogen mostly increases with temperature, although the first point at 25 °C does not fit this trend. We attribute this to the very small amounts of hydrogen produced at this temperature. This is close to the detection limit of our GC, which makes these measurements less accurate, thus overestimating the amount of hydrogen. Higher temperatures lead to an increasing carbon efficiency for CO, which is also observed at −0.95 V vs RHE. At −1.1 V vs RHE, the carbon efficiency not only toward CO but also toward HCOOH is increased at high temperatures. This increase seems mainly due to the fast decrease in CE of the C2+ products, which results in a relative increase in CE toward CO and HCOOH.

### 3.2 CO_2_ Concentration and Mass Transport

In all of the results discussed above, the temperature change is convoluted with a change in CO_2_ concentration in the electrolyte. This effect can be corrected for by performing partial pressure experiments in which the concentration of CO_2_ can be adjusted independently of the temperature. Figure S8 shows the effect of the bulk CO_2_ concentration on the FE at 25 °C. The chosen concentrations are equivalent to the maximum CO_2_ concentrations at 25, 40, 55, 70, and 85 °C in equilibrium with 1 atm of CO_2_. A lower CO_2_ bulk concentration leads to an increase in hydrogen evolution but not nearly as much as in Figure 1. With decreasing partial pressure, the FE toward methane increases, while the FEs for HCOOH and CO decrease. The FE toward C2+ products is not significantly affected by the changing CO_2_ concentration. These results mostly agree with the literature, in which either the partial pressure was changed^38,39^ or the local CO_2_ availability was determined.^40^ Other studies found that the ethylene activity can increase with slightly lowered partial pressures of CO_2_,^41^ in agreement with our observations.

In Figure S9, the concentration of CO_2_ was kept constant at different temperatures by changing the partial pressure accordingly. All measurements were performed with 14 mM of CO_2_ in the bulk. This concentration is equivalent to the concentration at 70 °C at 1 atm of CO_2_ as calculated with eqs 2 and 3. The trends observed here are very similar to the trends observed in Figure 1: there is an increase in selectivity toward hydrogen, a decrease toward methane formation, and an optimum in C2+ product formation. However, the FE for CO is slightly different as it now also seems to show an optimum at 55 °C. Furthermore, the HCOOH selectivity seems to be stable, except at the highest temperature. From these results we conclude that most trends in Figure 1 are caused directly by the increase in temperature and not indirectly by the decrease in CO_2_ solubility with increasing temperature. However, formic acid is an exception to this observation as the change in selectivity seems mostly governed by the changes in the CO_2_ bulk concentration, suggesting that formic acid is made in a different pathway from the other products. Moreover, the steadiness in CO selectivity appears to be due to the convolution of the enhancing effect of temperature and decreasing effect of the CO_2_ concentration.

The large drop in FE toward CO at 70 °C at −1.1 V vs RHE and the sharp increase in activity for HER at this temperature might indicate that there are some mass transport limitations at this temperature. We have shown before that efficient mass transport becomes more important at higher temperatures^16^ due to higher current densities and higher rates of the homogeneous reaction with OH^–^ generated at the surface.^42,43^ In our experiments, CO_2_ was bubbled through the cell at 40 sccm, which is the limit of our setup. This high flow rate, in combination with a PEEK-frit, is to facilitate mass transport and prevent CO_2_ depletion,^13,26^ but it might not be sufficient. When the mass transport boundary layer is not optimized, this can influence the selectivities significantly.^26^ To check whether an unoptimized mass transport boundary layer is involved in the observed trends, we have performed some experiments with enhanced mass transport by stirring the electrolyte with a small stirring bar in the cathode chamber. Figure S10 shows that all trends observed in Figure 1 do not significantly change when mass transport is improved. This indicates that the trends observed in this study are not caused by insufficient mass transport due to un unoptimized boundary layer. Local pH on the other hand is not influenced significantly by better convection^44^ and could still cause the changes in selectivity as will be discussed later.

Comparing our trends to the trends observed by Ahn et al.,^21^ their study shows a similar decrease in the selectivity toward methane and an increase toward hydrogen with increasing temperature, although in our case, hydrogen selectivity only increases at the highest temperatures (from 48 °C onward, which was not included in their work, which was limited to 42 °C). They also observe an optimum in ethylene selectivity but at lower temperature (22 °C). Their trends in FE toward CO and HCOOH are not very distinct, although the activity of CO formation clearly increases with temperature. We suspect that some of these differences are due to mass transport limitations, as in their experiments, the HER already dominates at 42 °C. Ahn et al. attributed some of their trends to the temperature dependence of the solubility of CO_2_, but our experiments show that this effect cannot explain most of the trends observed.

### 3.3 First Regime: CO Coverage

To assess whether the observed trends in the first temperature regime (i.e., below 48 °C) could be related to a temperature-dependent CO coverage on the copper electrode surface, we have performed in situ surface-enhanced Raman Spectroscopy (SERS) experiments. SERS is ideally suited to study CO2RR on Cu electrodes due to the ability to probe reaction intermediates, electrolyte species, and the electrode surface simultaneously.^45^ It is inferred that, with increasing CO coverage, the rate of CO dimerization should be favored,^46−49^ and therefore, one expects the production of ethylene or ethanol to be related to the CO coverage. Zhan et al.^36^ suggested a qualitative SERS-based measure of (the potential dependence of) surface CO coverage, namely, the peak ratio between the Raman peaks at 360 cm^–1^ (assigned to the Cu–C vibration of *CO on Cu) and 280 cm^–1^ (assigned to the Cu–CO twisted rotation or bending vibration). They postulated that a decrease in the 280 cm^–1^ band intensity compared to the 360 cm^–1^ band is indicative of high *CO coverage because multiple *CO close together will frustrate the Cu–CO bending vibration.^29^

**Figure 3:**
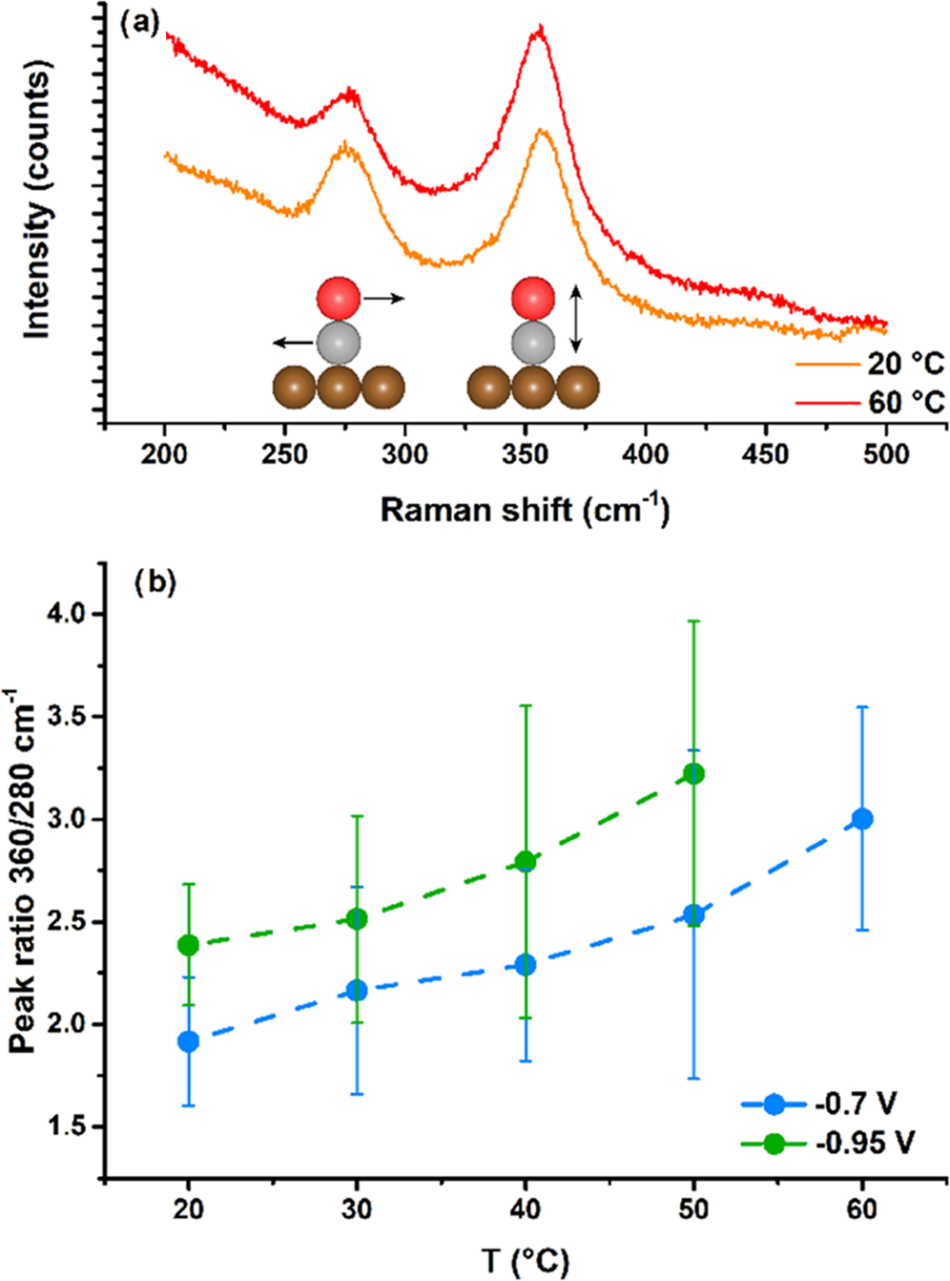
(a) Unprocessed Raman spectrum at 20 and 60 °C and −0.7 V vs RHE, representative for the other Raman spectra. (b) Ratio between the 360 and 280 cm^–1^ Raman peak areas on Cu in 0.1 M KHCO_3_ as a function of reaction temperature, at −0.7 and −0.95 V vs RHE. The error bars are composed of at least 4 spectra, and the dotted lines are a guide to the eye.

Figure 3a shows an example of an unprocessed Raman spectrum with these two peaks. Figure S11 gives more details how this data was processed to obtain the peak area ratios. Figure 3b shows that the coverage of *CO is affected not only by the potential but also by the temperature. With increasing temperature, the peak ratio, and the coverage of CO, increases both at −0.7 and −0.95 V vs RHE, which could explain the increase in C2+ products in the first regime. The literature suggests that the coverage at −0.95 is higher than at −0.7 V, which we also observe at all temperatures.^36^ In the SI and Figures S12–S16, the Raman data and analysis are discussed in more detail. From this analysis of the Raman spectra, we conclude an increasing trend in CO coverage with temperature, independently of the exact methods used to analyze the data. With our dataset, it is only possible to make a qualitative statement, but we consider this sufficient for the purpose of this research.

As adsorption is an exothermic process, we would expect the coverage to decrease with temperature based on a simple Langmuir isotherm (eq 4), as the equilibrium constant *K* should decrease with temperature according to the van ‘t Hoff equation. However, the local CO pressure can increase with increasing temperature because the rate of the CO_2_ reduction to CO production becomes higher at higher temperature. This would lead to a higher local CO concentration near the Cu surface^50^ and consequently the CO coverage.
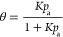
4where θ is the coverage, *K* is the equilibrium constant, and *p*_a_ is the (local) pressure or concentration.

Additionally, it has been proposed that significant amounts of CO_2_ are absorbed on the surface, thereby blocking part of the surface.^41,51^ It could be that due to the lower CO_2_ concentration, in combination with more active reduction, less adsorbed CO_2_ (*CO_2_) is blocking part of the Cu surface at elevated temperatures. Therefore, more CO can adsorb at the catalyst surface, and thereby, C–C coupling is facilitated when the temperature increases in the first regime.^41^

From eq 4, one would expect the CO coverage should decrease during CO reduction as both *K* and *p*_a_ would decrease with temperature because CO is not generated in situ and its solubility decreases with temperature. Thus, the temperature trend for CO reduction is expected to be different than for CO_2_ reduction, if the CO coverage is an important factor. Figures S17 and S18 show that for CO reduction indeed there is no optimum between 20 and 70 °C for both the C2+ product selectivity and activity. The FE toward ethylene decreases slowly up to 50 °C and then more rapidly from 50 to 70 °C. This is accompanied by a stable activity toward ethylene up to 50 °C, which starts to decrease at higher temperatures. These results confirm that the CO coverage is an important parameter affecting the C2+ formation rate. The increasing CO coverage with temperature during CO2RR thus provides a plausible explanation for the increase in C2+ products with increasing temperature during CO2RR.

### 3.4 Kinetics and Local pH

Although the coverage of CO can be linked to the selectivity trends observed in the first regime, the temperature dependence of CO_2_ reduction is a complicated interplay of direct and indirect effects.^16^ Temperature has a direct effect on the kinetics of the reactions via the Arrhenius equation. As the reaction pathways toward different products have different activation energies (*E*_a_), the changes in kinetics for each product should result in different selectivities. We have shown previously that, on a gold electrode, the (apparent) *E*_a_ for the CO2RR toward CO is higher than for the HER, which results in an increase in CO selectivity with increasing temperature.^16^ With our dataset, it is difficult to determine reliable apparent activation energies of the different products on copper (see the SI and Figures S19–S20 for a further discussion). Only for HER, a relatively accurate value could be determined, although this value is significantly lower than the value determined by Zong et al. (38 ± 2 kJ/mol vs ∼60 kJ/mol, respectively).^24^ They also show that methane has a significant lower apparent activation energy compared to both CO and ethylene. These results indicate that kinetics could give an explanation for the trends observed as methane selectivity decreases, while hydrogen selectivity increases with temperature. However, it is difficult to obtain accurate experimental data on the activation energies for CO2RR on copper due to the large variety of products and presumably even more difficult to interpret them.

An indirect effect of the temperature rise is an increase in local pH, as a result of the higher current densities related to a higher OH^–^ production. This local pH increase will certainly influence the reaction rates and the corresponding product distribution. Higher local pH is beneficial for ethylene selectivity, while it inhibits both HER and methane formation.^40,52,53^ However, there is an optimum in the influence of the local pH, and when increased too much, the selectivity toward ethylene decreases again.^53,54^ When the local pH is too high, the OH^–^ near the surface reacts with CO_2_ to form bicarbonate via the homogeneous reaction,^42,43^ lowering the CO_2_ concentration near the surface, which causes the FE for hydrogen to increase.^54^

In this perspective, the trend in total CO_2_ consumption at different potentials is interesting (Figure 2b). At lower potentials, the current densities are lower and the optimum in CO_2_ consumption shifts to higher temperatures. This suggests that at a certain point the local CO_2_ availability will be limited due to a high local pH. Moreover, the total CO_2_ consumption at 70 °C is higher at −0.95 V than at −1.1 V, indicating that a too high local pH can be a factor in the second regime. However, at −0.95 V, the HER dominates the FE at 70 °C, even though significant amounts of CO_2_ are still being consumed. Furthermore, the total current density at −1.1 V slightly decreases at the start of the second regime (Figure 2a), so it is unlikely that the local pH increases in this region. However, a similar local pH might have a larger effect as there is a lower bulk concentration of CO_2_. Moreover, even at −0.7 V vs RHE, the activity of CO2RR decreases with increasing temperature, although the total current density is significantly lower than at −1.1 V vs RHE. This indicates that the increase in FE for HER under these conditions is not solely related to a lack of CO_2_ due to a too high local pH. Furthermore, the trends in product distribution are similar at both potentials, as also at −0.95 V CO_2_ is mostly converted toward CO and less C_2_H_4_ is produced at the highest temperatures (Figure S4). This indicates that the decrease in C2+ products in the second regime is likely not exclusively due to the changes in the local pH.

Summarizing, the trends observed in the first regime between 18 and 48 °C can probably be explained by a combination of CO coverage, differences in kinetics, and local pH. With higher temperatures, the rate of CO2RR to CO increases, and hence, the CO coverage increases, boosting the C2+ formation. The local pH also increases, which is less favorable for methane formation and better for ethylene. Moreover, the activation energy for methane seems to be smaller than for the other CO2RR products, although activation energies are difficult to determine and interpret. The lower CO_2_ bulk concentration with increasing temperature cannot be linked to most observed selectivity trends. However, it is probably the main reason for the decrease in formic acid selectivity. On the other hand, this decrease might also indicate a lower coverage of the OCHO* intermediate, which has been suggested as a key intermediate for HCOOH formation.^55^

### 3.5 Second Regime: Surface Change

**Figure 4:**
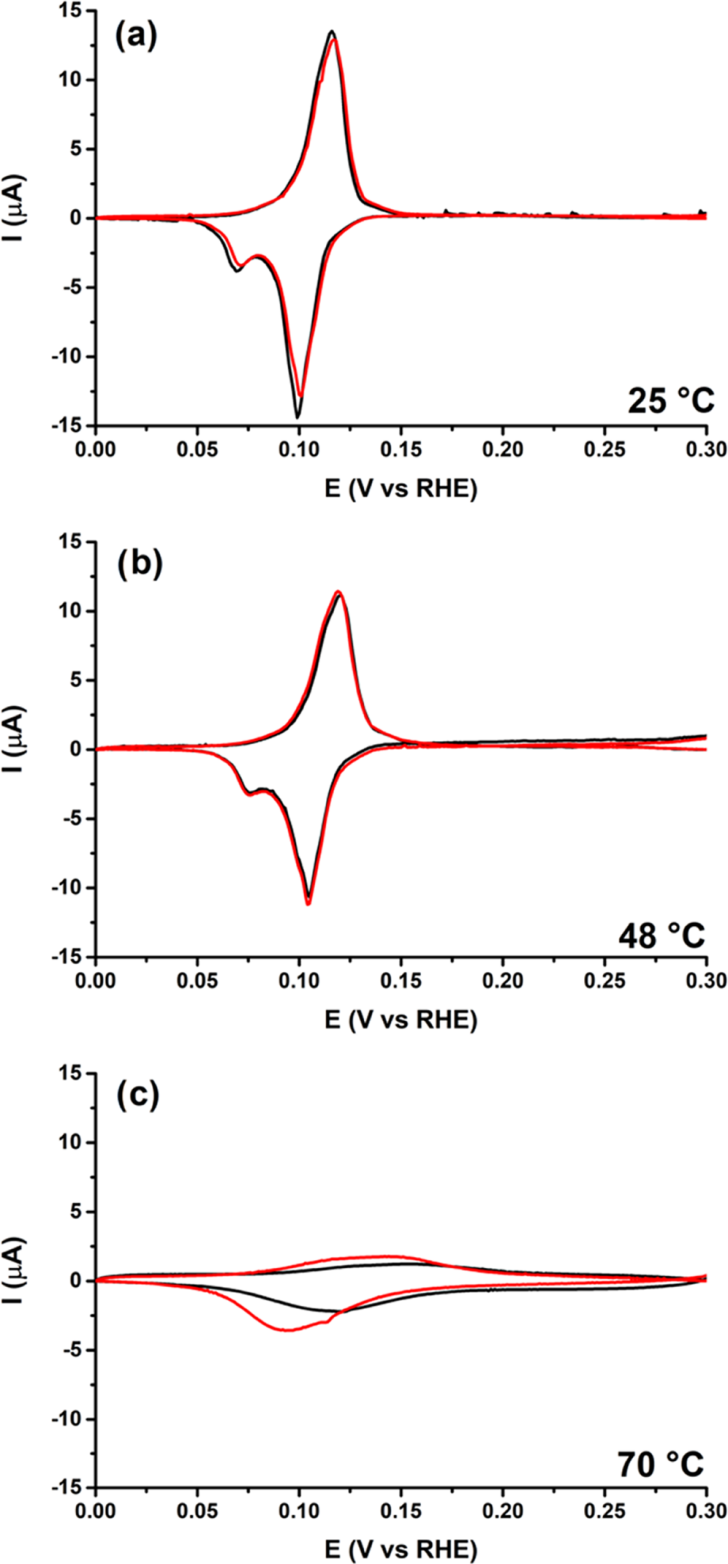
Pb UPD CVs from 0.0 to 0.3 V vs RHE at 5 mV/s in 0.1 M NaClO_4_ + 1 mM NaCl + 2 mM PbClO_4_ after CO2RR for 20 min at −1.1 V vs RHE in 0.1 M KHCO_3_ at (a) 25 °C, (b) 48 °C, and (c) 70 °C. The red and black line represent two different measurements to illustrate reproducibility.

In the second regime, the CO2RR activity decreases significantly at higher temperatures and HER dominates. This seems not due to the lower bulk CO_2_ concentration as shown with partial pressure experiments; however, a too high local pH probably does have some contribution here. To check if this apparent deactivation might also be related to a change in the copper surface at elevated temperatures, we have performed electrochemical surface characterization experiments. Figures 4, S21, and S22 show cyclic voltammograms (CVs) of Pb underpotential deposition (UPD) on the Cu electrode after CO_2_ reduction at different temperatures. Pb UPD is known to be sensitive to the structure of the Cu surface.^37^ The blank CV (Figure S21a) was measured before CO2RR but after the polishing procedure and shows two major peaks in the reduction scan. The peak at 0.07 V vs RHE is attributed to Cu(100) sites and the peak at 0.10 V vs RHE to Cu(111) sites.^37^ In the oxidation scan, the peaks are more convoluted as the Cu(100) peak is less reversible compared to the Cu(111) peak. The shape of the CV stays similar to the blank up to 48 °C, although the peaks shift slightly positively and become gradually smaller. The smaller peak areas indicate that the surface smoothens at higher temperatures, as the Pb UPD can also be used qualitatively to measure the electrochemical surface area (ECSA).^37^ However, as the shape of the CVs stays the same, no major surface changes are likely to take place up to 48 °C. On the other hand, when the temperature is increased beyond 48 °C, the Pb UPD CV changes drastically. At 55 °C, the Cu(100) peak seems to disappear and the Cu(111) broadens. However, the exact change in the CV is not reproducible, whereas it was very reproducible at the lower temperatures (Figure S21e). At 70 °C, the changes are even more pronounced as the Pb UPD peaks almost completely disappear.

From these observations, it is clear that the copper surface undergoes substantial changes at high temperature, but it is unclear what these changes entail exactly as the Pb UPD peaks are not observed anymore. Carbon might deposit on the copper surface at higher temperatures, comparable to surface chemistry reactions taking place in thermocatalytic processes, although for CO_2_ reduction to methanol on Cu, coking is normally not a significant cause of deactivation.^56^ On the other hand, it has been claimed that coking can occur during electrochemical CO_2_ reduction as well.^57,58^ This could explain the broadening and eventually disappearance of the Pb UPD peaks. However, EDX measurements (see Figure S24) show that, although some carbon can be detected at the copper surface, the composition of the Cu electrode does not change after CO2RR at different temperatures. The carbon can be due to the SEM beam itself,^59^ and as the amount of carbon does not change with different samples, we assume that no deposit is formed during CO2RR. The observed oxygen is most likely due to the oxidation of the copper surface during the transfer through air from the electrochemical cell to SEM-EDX. However, a recent theoretical study suggests that oxygen diffuses faster from the bulk to the surface at higher temperatures causing the residual Cu_2_O to be reduced quicker,^60^ which might change the nature of the copper catalyst and its selectivity. Similar to the Pb UPD measurements, the SEM images indicated that the surface becomes smoother at higher temperatures (Figure S25).

Double layer capacitance studies have been performed to confirm this smoothening in a quantitative manner. This technique gives an indication of the roughness factor and these measurements confirm that at higher temperatures the surface becomes less rough (Figures S26 and S27). Interestingly, it seems that this difference is mainly due to a roughening of the surface after CO2RR at 25 °C, while at 40 and 55 °C, the surface roughness does not significantly change after CO2RR. At 70 °C, the surface actually seems to become smoother during electrolysis.

Recently, the group of Buonsanti^61,62^ showed that Cu dissolves under negative applied potentials, with subsequent redeposition on the electrode roughening the electrocatalyst surface. Based on their results, we hypothesize that this dissolution can be promoted by small amounts of oxygen in the system as they show that the dissolution is enhanced in air.^62^ Even though CO_2_ is being purged throughout the experiment, we cannot guarantee that the cell is completely oxygen-free. At higher temperatures, surface diffusion of the copper atoms is expected to be enhanced. This process might be facilitated by CO produced on the surface, equivalent to the increased Cu surface mobility with CO in gas phase.^63^ Since the CO coverage increases with increasing temperature, more CO is available to smoothen the surface. A combination of these processes could explain why smoother copper surfaces are observed at higher temperatures. A more flat surface indicates that there are fewer defects and the surface is more ordered. Such surfaces tend to mainly produce hydrogen, whereas hydrocarbons tend to be formed at the defect sites.^64^ A smoother surface at high temperature could thus be one of the reasons for the increase in hydrogen evolution.

Thus, the copper surface changes with temperature, both gradually over the entire temperature range and rapidly at temperatures higher than 48 °C. However, it is unknown what these changes are exactly and how they are related to the trends in selectivity observed.

### 3.6 Reversibility of Temperature Effect

**Figure 5:**
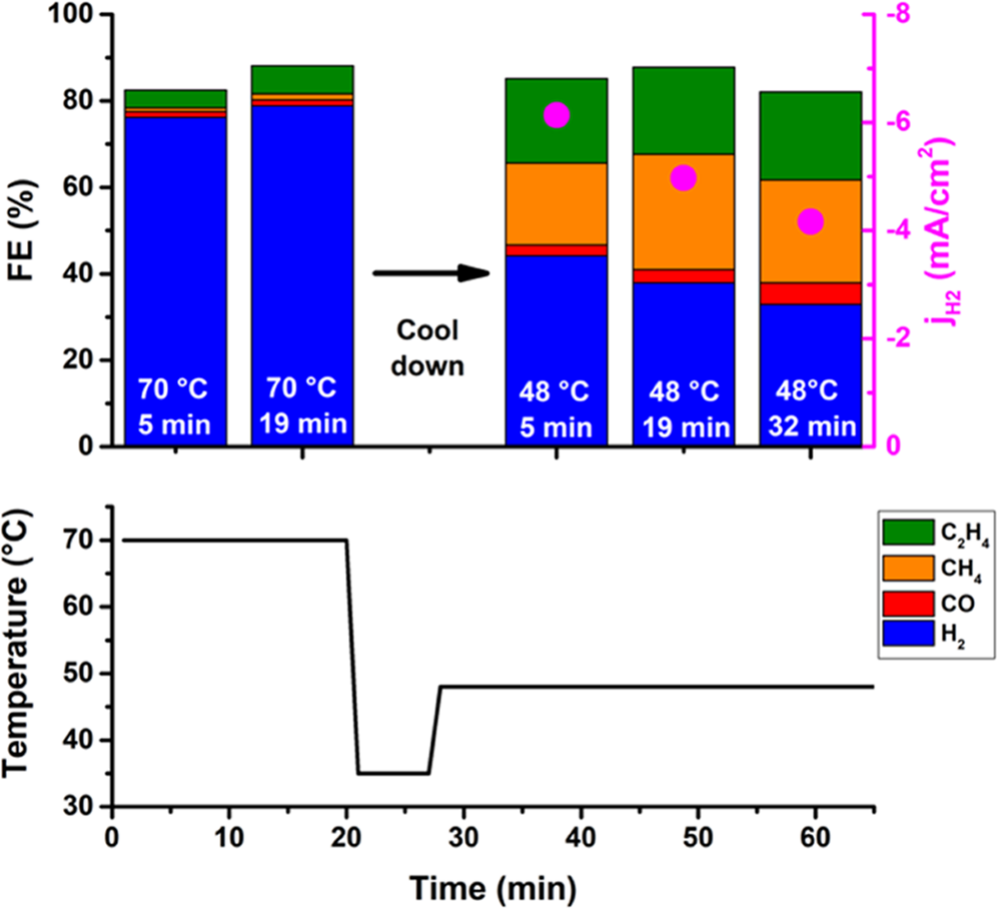
Reversibility experiments; Faradaic efficiency (FE) of the gaseous products of CO2RR at −1.1 V vs RHE at 70 °C after 5 and 19 min and after cooling down at 5, 19, and 32 min at 48 °C. The current density of HER is shown with the magenta dots (at 70 °C, these are too large and fall of the scale). The lower panel shows the temperature profile of the water bath.

At high temperature, copper mainly produces hydrogen, whereas the optimum in the ethylene production is at 48 °C. However, this change in selectivity can be reverted when the same Cu electrode is cooled down again from 70 to 48 °C, as shown in Figure 5. This can be compared to the control experiment at 48 °C in Figure S28. Even when the catalyst has experienced CO2RR at high temperature and its surface has changed, it can still produce comparable amounts of ethylene and methane as when the catalyst remains at maximum 48 °C (see Figure S28). Interestingly, the selectivity keeps changing with time after the electrode is cooled down from 70 to 48 °C; especially, the hydrogen activity decreases with time. The changes in selectivity are thus reversible although not instantaneous. Remarkably, the changes seen in the Pb UPD are not reversible. If the Cu electrode is first used at 70 °C and then CO2RR is performed at 48 °C, the Pb UPD is still comparable to the Pb UPD of the electrode which was only used at 70 °C (Figure S23). This observation complicates our interpretation: even though the change in the copper surface and the optimum in activity coincide, this might be just a coincidence, and the changes on the surface of the copper might not be the cause of the deactivation of the CO2RR in the second regime.

## 4 Conclusions

In this work, we have investigated the effect of the reaction temperature between 18 and 70 °C on the CO_2_ reduction on copper. We observe that it is beneficial to work at elevated temperatures and the optimal temperature for CO2RR electrolysis would be ∼48 °C. Different products respond differently to the reaction temperature. The selectivity of methane and formic acid decreases with increasing reaction temperature, while the C2+ products show an optimum in both selectivity and activity around 48 °C. At high temperature (e.g., 70 °C), the Faradaic efficiency for hydrogen evolution dominates, while CO_2_ is mostly reduced to CO. The temperature range can thus be divided in two regimes: a first regime from 18 up to ∼48 °C, in which C2+ production increases and hydrogen evolution selectivity stays fairly constant, and then a second, high-temperature regime from 48 to 70 °C, in which hydrogen starts to dominate and the activity of CO2RR decreases. Our results show that, for industrial applications at elevated reaction temperatures, it will be crucial to find strategies to limit HER and that temperature is a crucial parameter to consider for CO2RR, also in GDE setups. Furthermore, we show that CO reduction does not experience an optimum in C2+ products, which can have implications if one would use a cascade reaction to produce C2+ products at elevated temperatures.

The observed trends are not simply due to the balance of reaction kinetics and CO_2_ solubility. Although kinetics will certainly play a role in the first regime, an increase in both the CO coverage (as determined with Raman spectroscopy) and the local pH will result in a change in the selectivities. For the second regime it is more difficult to determine the exact causes of the decrease in CO2RR activity, although with partial pressure experiments, we showed that this is not due to the decreasing CO_2_ bulk concentration. Pb UPD and double layer capacitance studies show substantial structural changes in the copper surface at these high temperatures, which may be related to the changing selectivities. Moreover, this decrease could be partially due to a too high local pH. Future experiments will have to consider using a GDE setup to improve mass transport and see if the onset of the second regime is intrinsic to copper or due to the system used. Moreover, elevated pressures could be used to increase the solubility of CO_2_, which allows a more in depth study of the effect of pressure and temperature, especially from the second, high-temperature regime onward.

## Supporting Information Available

Additional experimental results including selectivity trends at −0.95 and −0.7 V vs RHE, partial pressure experiments, extra information about the data processing of the Raman experiments, CO reduction experimental data with temperature, Arrhenius plots, additional Pb UPD cyclic voltammograms at different temperatures, SEM-EDX data, and double layer capacitance results (PDF)

## Abbreviations

CO2RR: electrochemical CO_2_ reduction
CE: carbon efficiency
CV: cyclic voltammetry
FE: Faradaic efficiency
GDE: gas diffusion electrode
GC: gas chromatograph
HER: hydrogen evolution reaction
HPLC: high-performance liquid chromatography
RHE: reversible hydrogen electrode
