# Supplementary material for: How Temperature Affects the Selectivity of the Electrochemical CO2 Reduction on Copper
Source: ACS Catal. 2023 Jun 1;13(12):8080–91. doi: 10.1021/acscatal.3c00706 (PMC10278069; doi:10.1021/acscatal.3c00706)
Supplement: Supplementary file 1 — cs3c00706_si_001.pdf [file cs3c00706_si_001.pdf]

Supporting Information to

How temperature affects the selectivity of the electrochemical CO_2 reduction on copper

Rafaël E. Vos[a], Kees E. Kolmeijer[a], Thimo S. Jacobs[b], Ward van der Stam[b], Bert M. Weckhuysen[b], Marc T.M. Koper[a]*

[a] Leiden Institute of Chemistry, Leiden University, P.O.Box 9502, 2300 RA Leiden, The Netherlands

[b] Inorganic Chemistry and Catalysis group, Debye Institute for Nanomaterials Science and Institute for Sustainable and Circular Chemistry, Utrecht University, Universiteitsweg 99, 3584 CG, Utrecht, The Netherlands

*E-mail: m.koper@chem.leidenuniv.nl

Table of content:

Photo and scheme of the H-cell and jacket	2
CO_2 reduction selectivity and activity at -1.1V vs. RHE	3
CO_2 reduction selectivity and activity at -0.95V vs. RHE	4
CO_2 reduction selectivity and activity at -0.7V vs. RHE	7
Partial pressure experiments	8
Stirring experiments	10
Raman Spectroscopy experiments	11
CO reduction experiments	15
Activation energies	16
Pb UPD	17
SEM-EDX	20
Double layer capacitance	22
Reversibility experiment, control experiment	23

Photo and scheme of the H-cell and jacket

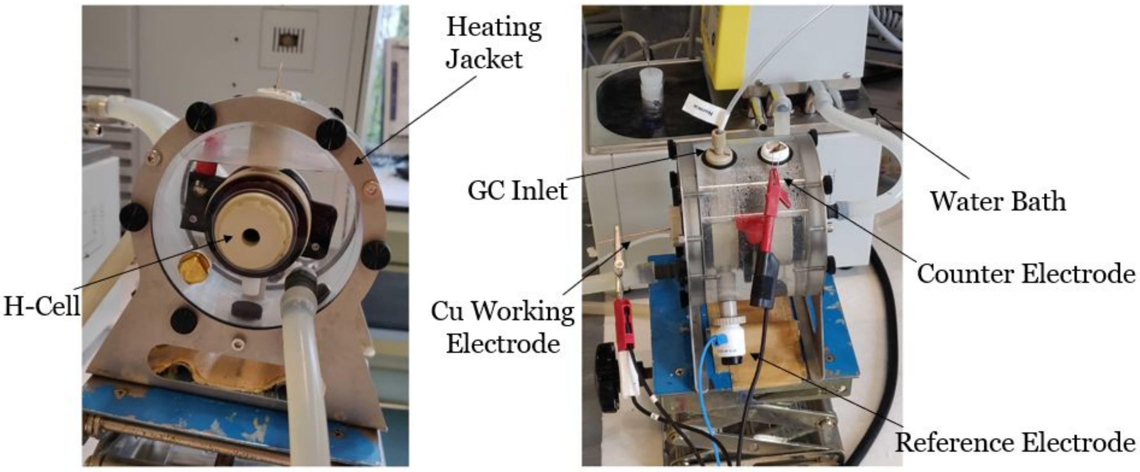

Figure S1: Photos from the H-cell surrounded by the water jacket used to control the temperature

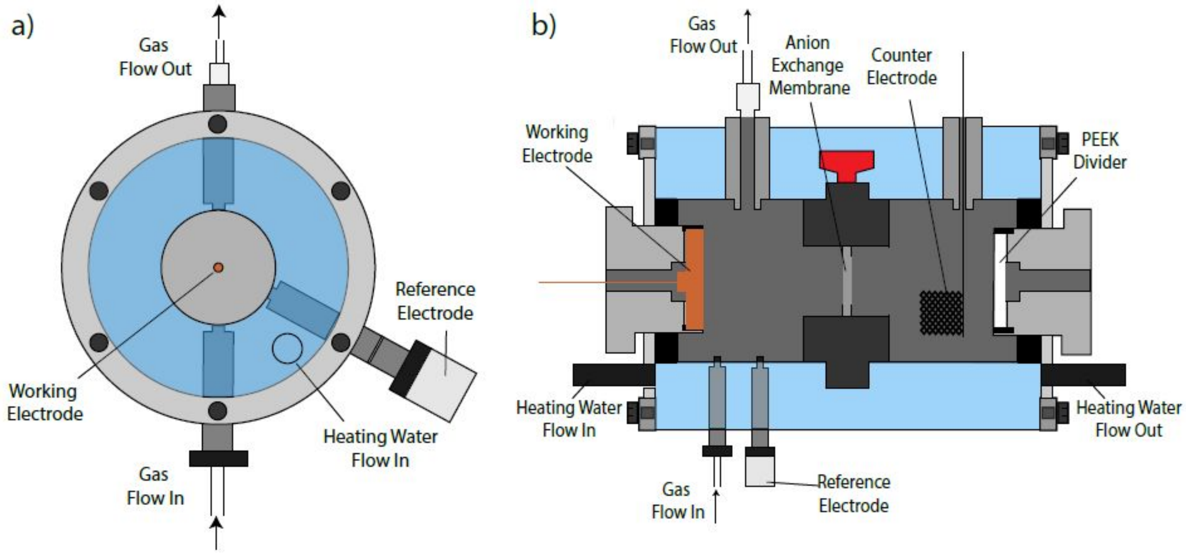

Figure S2: Schematics of the H-cell surrounded by the water jacket used to control the temperature. a) Frontal-section of the cathodic chamber and (b) cross-section of the electrochemical H-cell with heating jacket

CO_2 reduction selectivity and activity at -1.1V vs. RHE

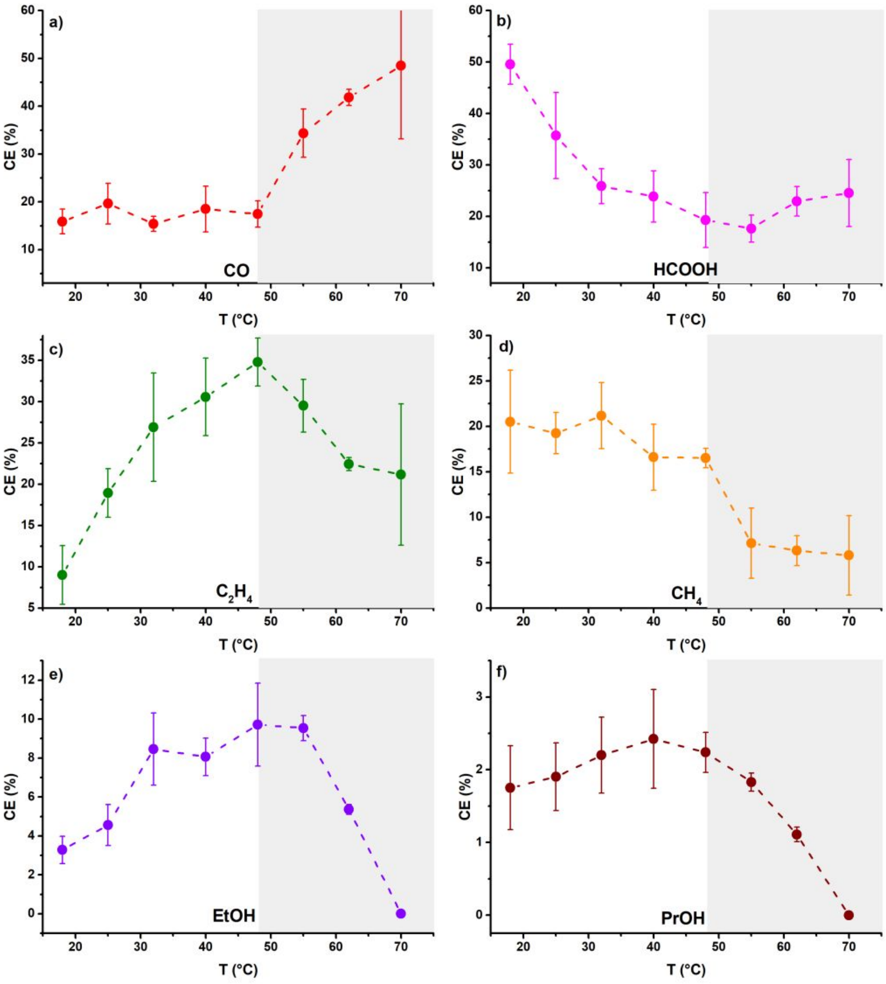

Figure S3 Carbon Efficiency of CO2RR at different reaction temperatures in 0.1 M KHCO3 at -1.1 V vs. RHE for a) CO b) formic acid c) ethylene d) methane e) ethanol, and f) 1-propanol. The error bars are determined from at least 3 separate experiments. The gray background indicates the second regime and the dotted lines are a guide to the eye.

Table 1: Faradaic and Carbon efficiency for CO2RR at different temperatures at -1.1V vs. RHE

-1.1 V	FE/CE (%)							
	18 °C	25 °C	32 °C	40 °C	48 °C	55 °C	62 °C	70 °C
H_2	29.4 / -	27 / -	28.7 / -	29.3 / -	41.0 / -	48.7 / -	62.3 / -	84.0 / -
CO	4.7 / 15.9	6.4 / 19.6	5.3 / 15.4	5.9 / 18.5	4.5 / 17.5	7.7 / 34.4	5.9 / 41.9	1.9 / 48.5
CH_4	21.5 / 20.5	17.9 / 19.3	20.1 / 21.2	18.1 / 16.6	13.5 / 16.5	6.6 / 7.2	4.2 / 6.3	1.2 / 5.8
C_2H	5.9 / 9.0	13.5 / 18.9	18.8 / 26.9	21.1 / 30.6	21.9 / 34.8	16.6 / 29.5	9.9 / 22.4	2.2 / 21.2
HCOOH	13.3 / 49.6	11.3 / 35.7	9.9 / 25.9	6.5 / 23.9	4.7 / 19.3	3.5 / 17.6	3.7 / 22.9	1.2 / 24.5
EtOH	2.6 / 3.3	4.1 / 4.6	6.4 / 8.5	7.2 / 8.1	6.9 / 9.7	4.4 / 9.5	2.6 / 5.4	0.6 / 0.1
PrOH	1.4 / 1.8	1.8 / 1.9	1.7 / 2.2	2.1 / 2.4	1.6 / 2.2	0.9 / 1.8	0.5 / 1.1	0.0 / 0.0

CO_2 reduction selectivity and activity at -0.95V vs. RHE

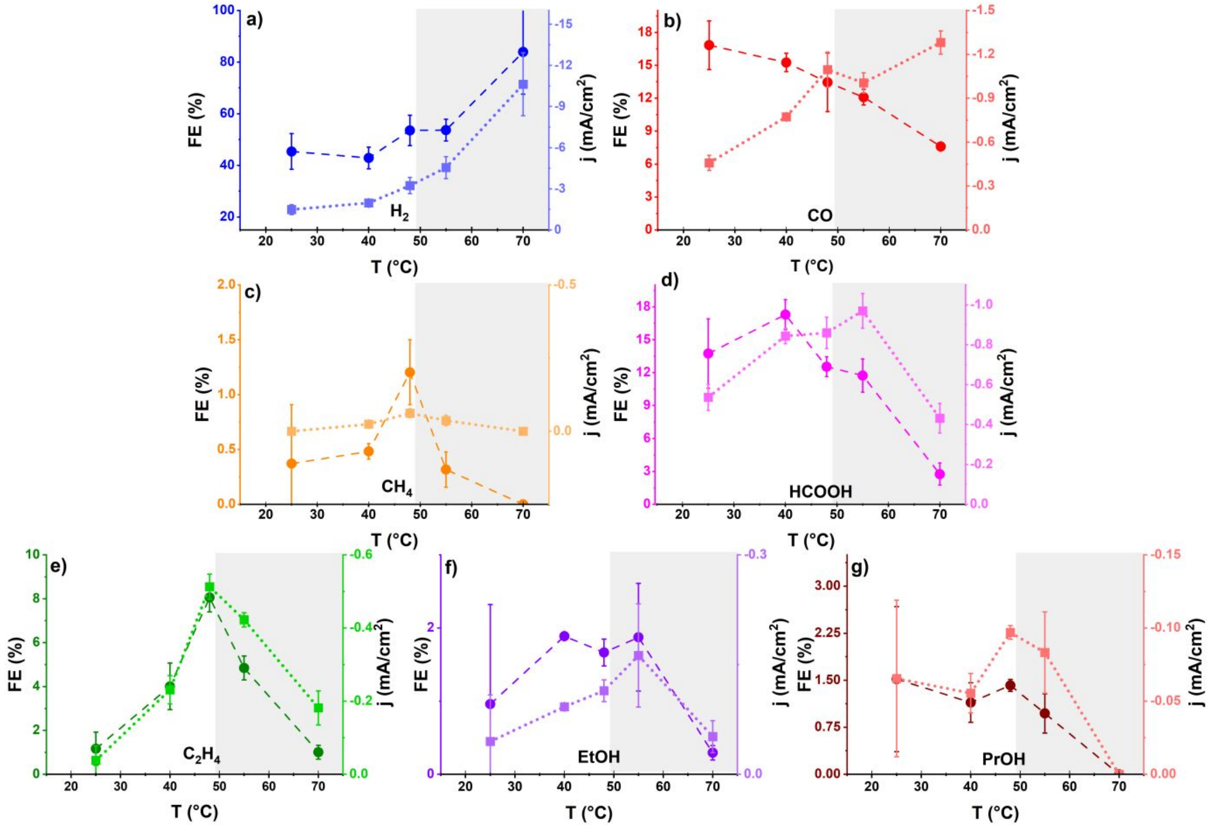

Figure S4: Faradaic efficiency (in dark circles) and partial current density (in light squares) of CO2RR at different temperatures in 0.1M $KHCO_3$ at -0.95V vs. RHE for a) hydrogen b) CO c) methane d) formic acid e) ethylene f) ethanol g) 1-propanol. Dotted lines are a guide to the eye and error bars are determined from at least 3 measurements.

Table 2: Faradaic and Carbon efficiency for CO2RR at different temperatures at -0.95V vs. RHE

-0.95 V	FE/CE (%)				
	25 °C	40 °C	48 °C	55 °C	70 °C
H_2	45.4 / -	42.9 / -	53.5 / -	53.7 / -	83.9 / -
CO	16.8 / 43.8	15.3 / 44.2	13.5 / 49.5	12.1 / 45.5	7.6 / 71.6
CH_4	0.4 / 0.1	0.5 / 0.3	1.2 / 0.7	0.3 / 0.4	0.0 / 0.0
C_2H	1.2 / 1.2	4.0 / 4.4	8.1 / 7.7	4.8 / 6.4	1.0 / 3.4
HCOOH	13.7 / 51.4	17.3 / 48.2	12.5 / 38.9	11.7 / 44.0	2.8 / 24.1
EtOH	1.0 / 1.4	1.9 / 1.8	1.7 / 1.7	1.9 / 2.5	0.3 / 1.0
PrOH	1.5 / 2.1	1.1 / 1.1	1.4 / 1.5	1.0 / 1.3	0.0 / 0.0

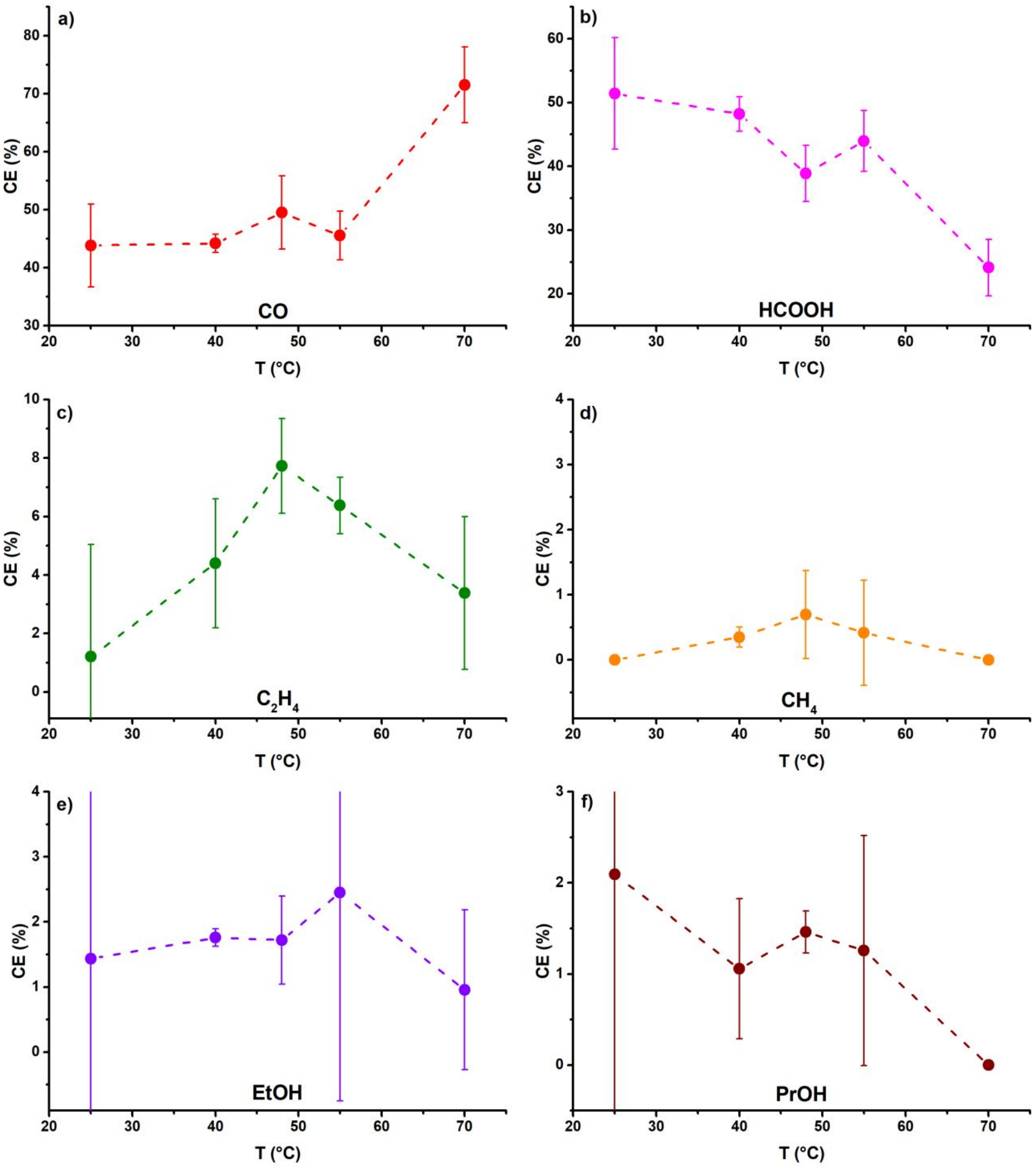

Figure S5: Carbon efficiency of CO2RR at different temperatures in 0.1 M $KHCO_3$ at -0.95 V vs. RHE for a) formic acid b) CO c) ethylene d) methane e) ethanol f) 1-propanol. Dotted lines are a guide to the eye and error bars are determined from at least 3 measurements.

CO_2 reduction selectivity and activity at -0.7V vs. RHE

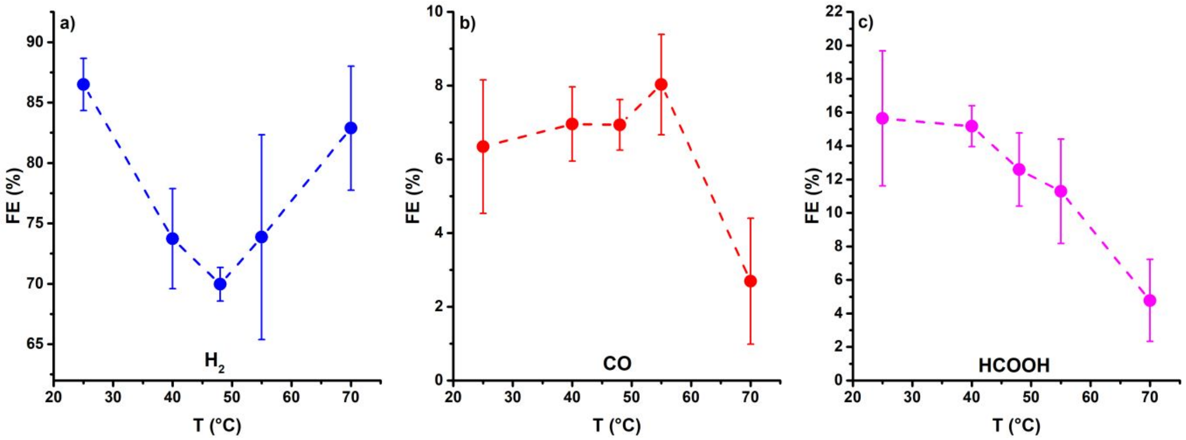

Figure S6: Faradaic efficiency of CO2RR at different temperatures in 0.1 M $KHCO_3$ at -0.7 V vs. RHE for a) hydrogen b) CO c) formic acid

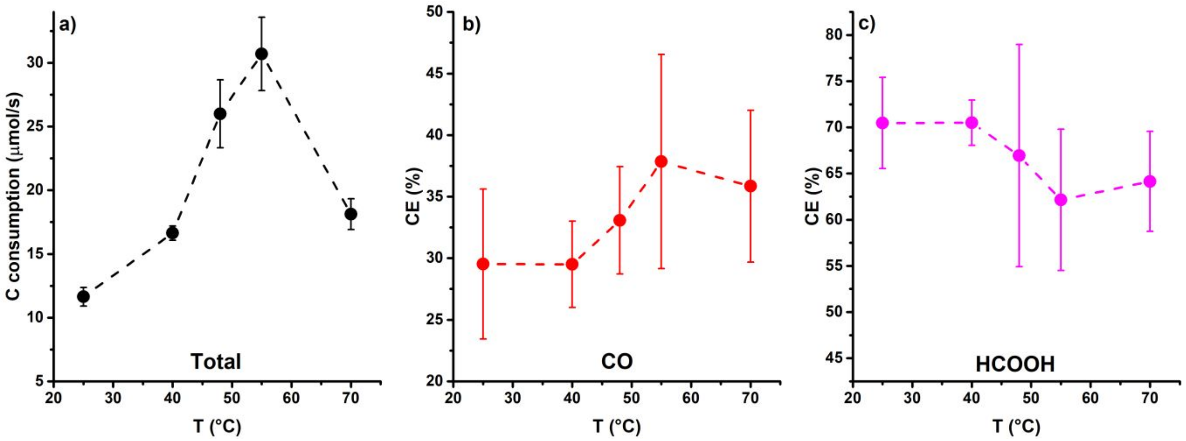

Figure S7: a) Total consumption of CO_2 of CO2RR at different temperatures in 0.1 M $KHCO_3$ at -0.7 V vs. RHE and the carbon efficiency towards b) CO and c) HCOOH. Dotted lines are a guide to the eye and error bars are determined from at least 3 measurements.

Table 3: Faradaic and Carbon efficiency for CO2RR at different temperatures at -0.7V vs. RHE

-0.70 V	FE/CE (%)				
	25 °C	40 °C	48 °C	55 °C	70 °C
H_2	86.5 / -	73.7 / -	70.0 / -	73.9 / -	82.9 / -
CO	6.3 / 29.5	7.0 / 29.5	6.9 / 33.1	8.0 / 37.8	2.7 / 35.9
HCOOH	15.6 / 70.5	15.2 / 70.5	12.6 / 66.9	11.3 / 62.2	4.8 / 64.1

Partial pressure experiments

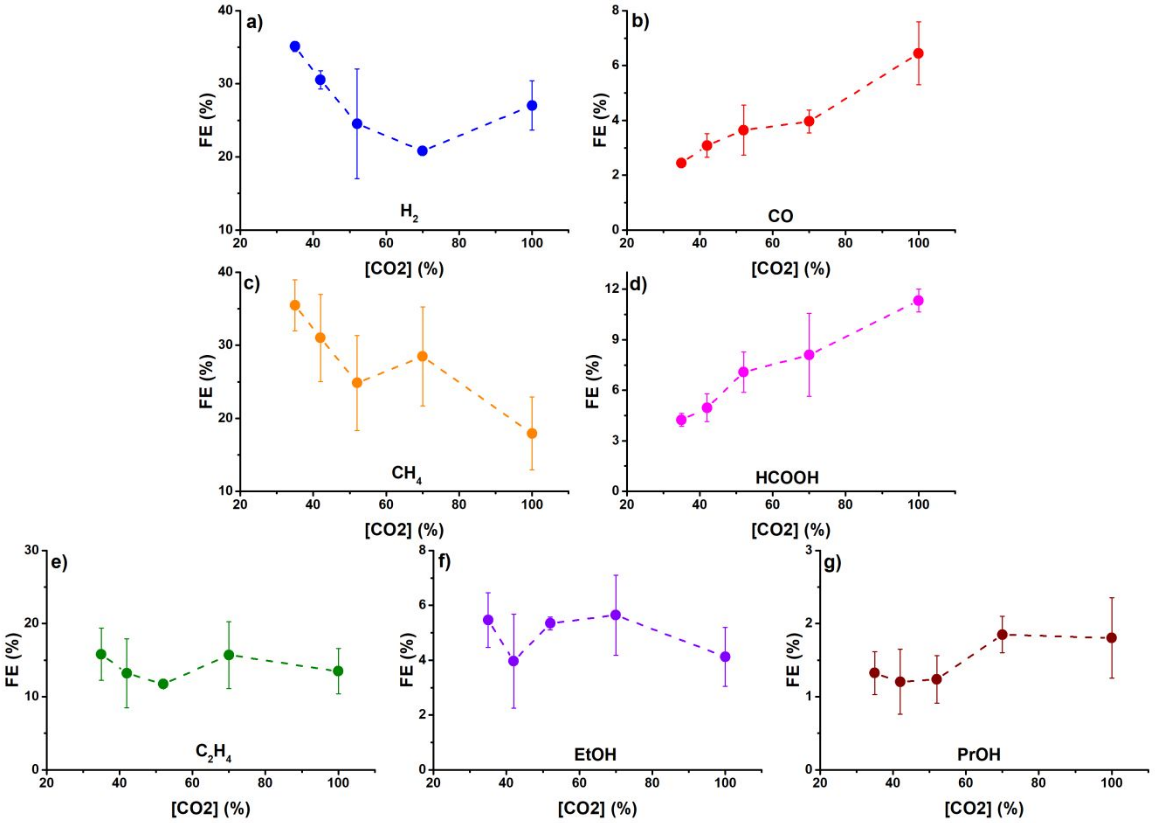

Figure S8: Faradaic efficiency of CO2RR at different CO_2 bulk concentrations by changing the partial pressure in 0.1 M $KHCO_3$ at -1.1 V vs. RHE for a) hydrogen b) CO c) methane d) formic acid e) ethylene f) ethanol g) 1-propanol. Dotted lines are a guide to the eye and error bars are determined from at least 3 measurements.

Table 4: Faradaic efficiency of CO2RR at different CO_2 bulk concentrations by changing the partial pressure in 0.1M $KHCO_3$ at -1.1V vs. RHE

Partial pressure	FE (%)				
[CO_2]	100 %	70 %	52 %	42 %	35 %
H_2	27.0	20.8	24.5	30.5	35.1
CO	6.4	4.0	3.6	3.1	2.4
CH_4	17.9	28.5	24.8	31.0	35.5
C_2H	13.5	15.7	11.7	13.2	15.8
HCOOH	11.3	8.1	7.1	5.0	4.2
EtOH	4.1	5.6	5.3	4.0	5.5
PrOH	1.8	1.8	1.2	1.2	1.3

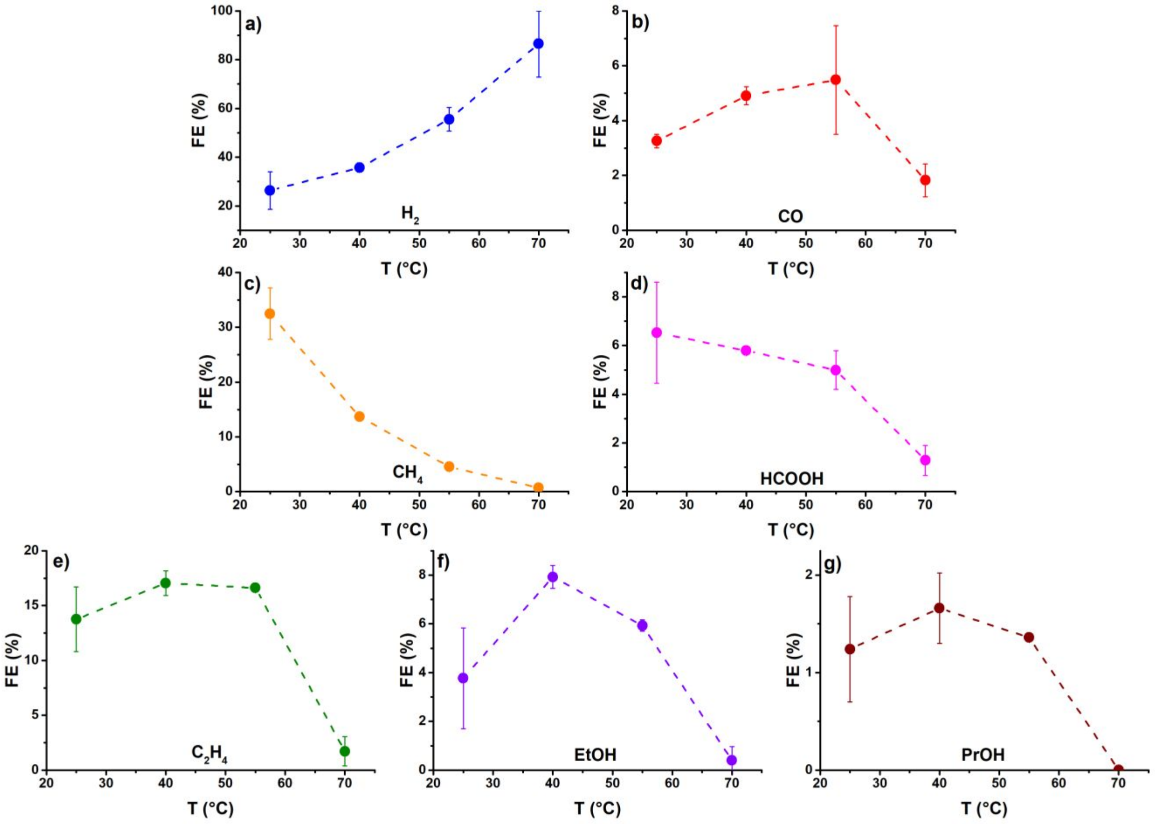

Figure S9: Faradaic efficiency of CO2RR at different temperatures with constant CO_2 bulk concentrations by changing the partial pressure in 0.1 M $KHCO_3$ at -1.1 V vs. RHE a) hydrogen b) CO c) methane d) formic acid e) ethylene f) ethanol, and g) 1-propanol. Dotted lines are a guide to the eye and error bars are determined from at least 3 measurements.

Table 5: Faradaic efficiency of CO2RR at different temperatures with constant CO_2 bulk concentrations by changing the partial pressure in 0.1M $KHCO_3$ at -1.1 V vs. RHE

$[CO_2]$ constant	FE (%)			
	25 °C	40 °C	55 °C	70 °C
H_2	26.3	35.7	55.5	86.5
CO	3.3	4.9	5.5	1.8
CH_4	32.5	13.7	4.5	0.7
C_2H	13.7	17.0	16.6	1.7
HCOOH	6.5	5.8	5.0	1.3
EtOH	3.8	7.9	5.9	0.4
PrOH	1.2	1.7	1.4	0.0

Stirring experiments

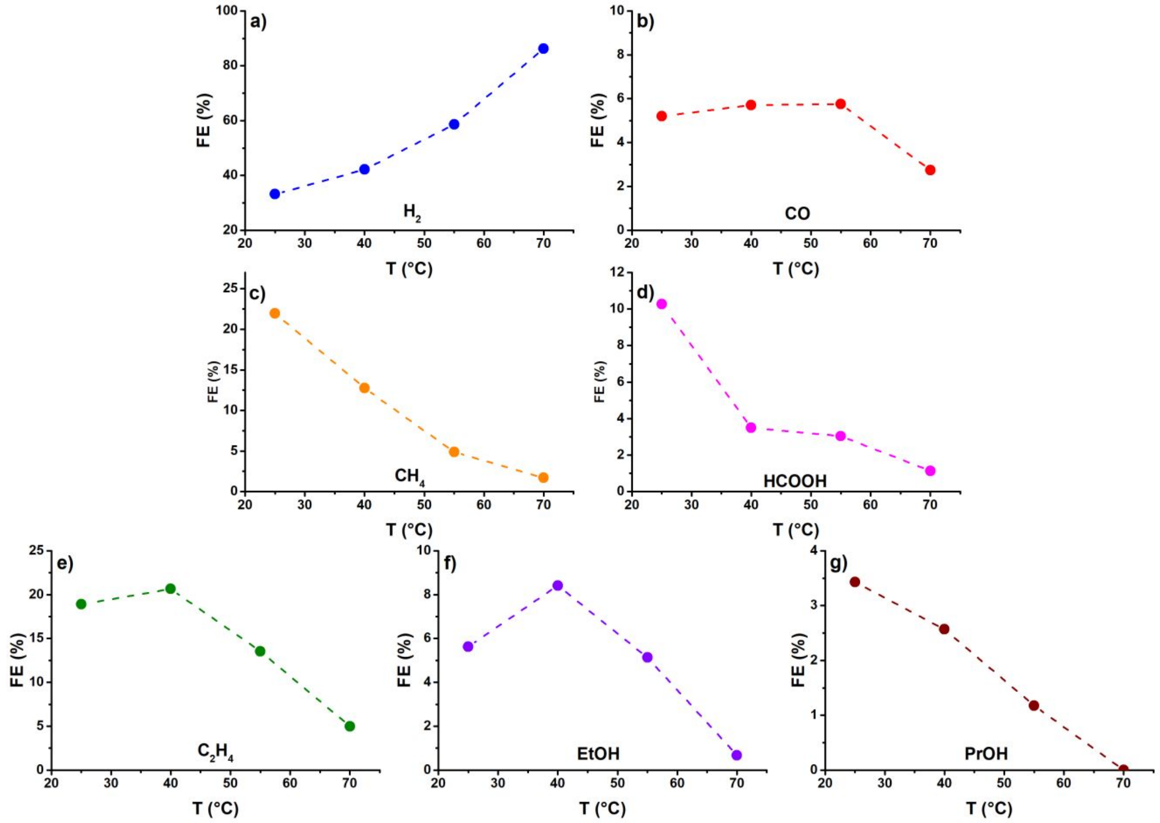

Figure S10: Faradaic efficiency of CO2RR at different temperatures while stirring with a stirring bar at 1000 rpm in 0.1 M $KHCO_3$ at -1.1 V vs. RHE for a) hydrogen b) CO c) methane d) formic acid e) ethylene f) ethanol, and g) 1-propanol. Dotted lines are a guide to the eye and error bars are determined from at least 3 measurements.

Table 6: Faradaic efficiency for CO2RR at different temperatures at -1.1V vs. RHE while stirring with at stirring bar at 1000 rpm

stirring		FE (%)		
	25 °C	40 °C	55 °C	70 °C
H_2	33.2	42.1	58.7	86.2
CO	5.2	5.7	5.8	2.7
CH_4	22.0	12.8	4.9	1.7
C_2H	18.9	20.6	13.5	5.0
HCOOH	10.3	3.5	3.0	1.1
EtOH	5.6	8.4	5.1	0.7
PrOH	3.4	2.6	1.2	0.0

Raman spectroscopy measurements

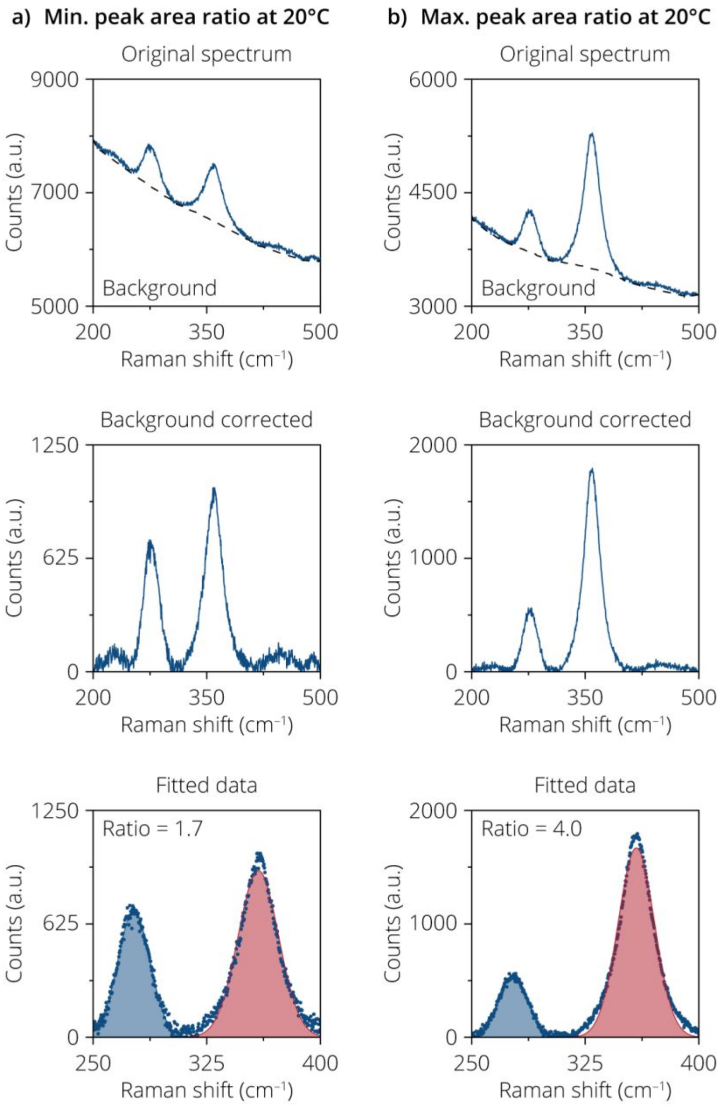

Figure S11: Example of the data processing of the Raman data. Panel a) shows an example with small peak area ratios and b) with large peak area ratios. From top to bottom the figure shows the original spectrum, the background corrected spectrum and then the data that is fitted with a Gaussian peak fitting procedure to calculate the area of the 280 cm^{-1} and 360 cm^{-1} peaks in blue and red, respectively.

Supporting Discussion: As mentioned in the experimental section, the obtained spectra were baseline corrected using the SNIP algorithm for background elimination. [1] After this background correction, a Gaussian fit was performed on the individual spectra to calculate the area of the 280 and 360 cm^{-1} features. The Raman shift window between 250 and 400 cm^{-1} was used for the fits, with a set boundary between the two peaks at 310 cm^{-1}. The ratio of the 360 and 280 cm^{-1} peaks areas was then used to determine the CO coverage qualitatively.

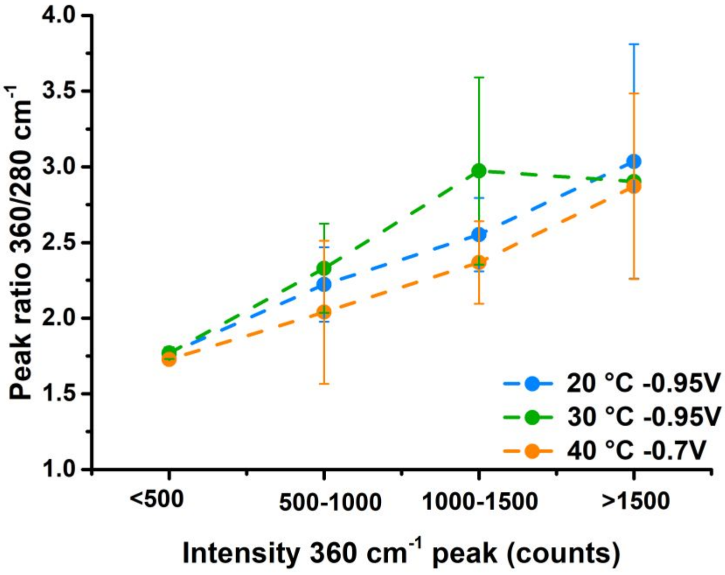

Figure S12: The ratio between 360 and 280 cm^{-1} peak areas on Cu in 0.1 M KHCO$_3$ vs. the peak intensity of the 360 cm^{-1} peak, which is used as a proxy for the intensity of the Raman spectra. Three different conditions are shown to illustrate that this trend is not a coincidence. It can be seen that the ratio depends on the intensity of the peaks: at higher intensities the peak area ratio increases.

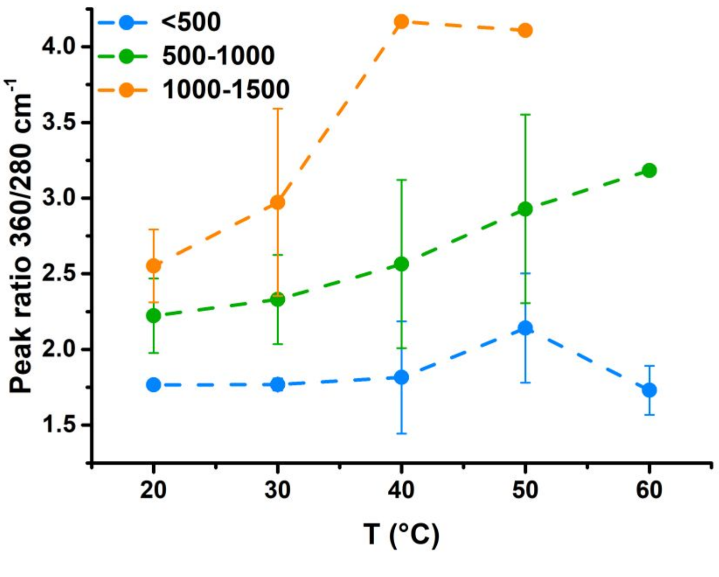

Figure S13: The ratio between 360 and 280 cm^{-1} peak area on Cu in 0.1 M KHCO$_3$ vs. temperature at -0.95 V vs. RHE. It can be seen that the intensity of the Raman spectra, here indicated by the peak intensity of the 360 cm^{-1} peak, influences the peak ratio. The effect of temperature on the peak ratio and thus the CO coverage is more pronounced with more intense peaks.

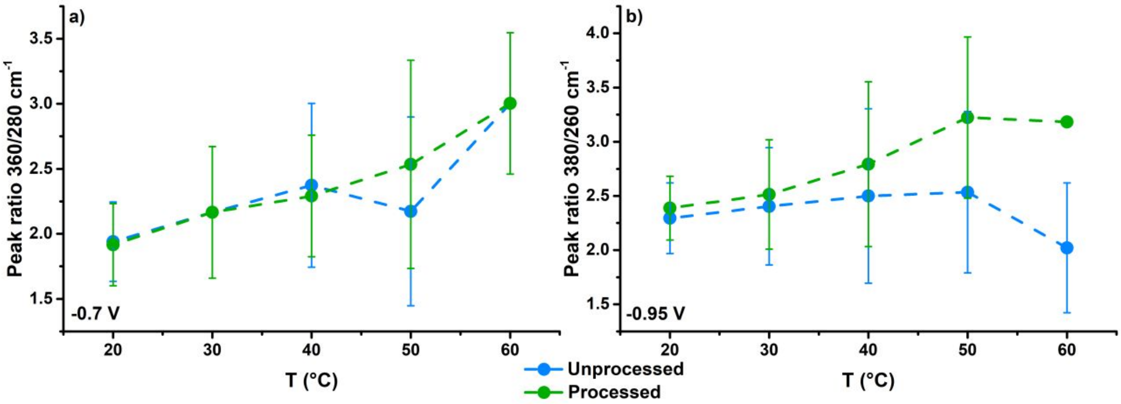

Figure S14: The ratio between 360 and 280 cm^{-1} peak areas on Cu in 0.1 M $KHCO_3$ vs. different temperatures at a) -0.7 V and b) -0.95 V. The blue line shows the average when all measured data points are taken into account, the green line shows the average when the peaks with low intensity are not taken into account.

Supporting discussion: In Figure 3b, the 280 and 360 cm^{-1} Raman features with the lowest intensities were not taken into account, because we observe that the peak area ratio also seems to depend on the intensity of these peaks, as can be seen in Figure S12. When the spectra are more intense, as indicated by the peak height of the 360 cm^{-1} feature, the peak area ratio increases as well. This might be caused by the inhomogeneity of the copper surface or by the extent to which the different bands respond to the surface enhancement. Therefore, we isolate the effect of temperature on the peak area ratio by only comparing peak area ratios of spectra with similar intensity, as can be seen in Figure S13. It can be seen that at all intensities, the CO coverage increases with temperature, although the trend becomes more pronounced when the peak area is larger. When all measured points are taken into account, the trends of Figure 3 are still observed, as can be seen in Figure S14. However, at -0.7 V the trend is less pronounced due to the data point at 50 °C, as the measurements at this temperature contained many low intensity peaks with a lower ratio. At -0.95 V the entire trend is less pronounced as there are more low intensity peaks in general. Additionally, the peak position is potential dependent due to the electrochemical Stark shift (Figures S15 and S16), but we find that this has negligible effect on the calculated peak ratios. From this analysis of the Raman spectra, we thus conclude an increasing trend in CO coverage with temperature, independently of the exact methods used to analyze the data.

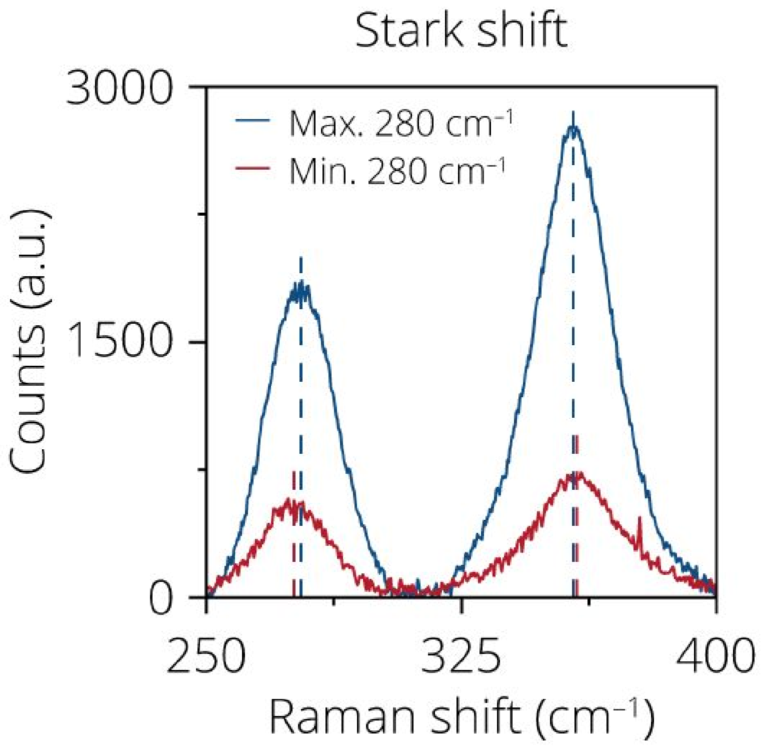

Figure S15: Raman spectra to highlight the changing 280 and 360 cm^{-1} peak position at different measurement spots on the same Cu electrode. The spectra were both measured at 20 °C and -0.7 vs. RHE. The blue line shows the highest 280 cm^{-1} peak position, while the orange line shows the lowest 280 cm^{-1} peak position. The dashed lines indicate the peak maxima obtained from the Gaussian fitting procedure

Supporting Discussion. The interfacial electric field that is generated at the electrode/electrolyte interface has implications for the energetics of the vibrational modes of the adsorbed intermediates. This is known as the vibrational Stark effect and has been researched for Raman spectroscopy during the CO2RR, where the adsorbed CO vibrations are known to show an electrochemical Stark shift due to the large dipole moment and sensitive interaction with the electrode surface. [2,3] Since the direction of the molecular vibration associated with the 280 cm^{-1} (assigned to the restricted rotation vibration of adsorbed CO) and 360 cm^{-1} (assigned to the Cu–Co stretching vibration) Raman bands with respect to the electric field is different, the expected Stark effect for these two vibrations should also be different. The molecular vibration associated with the 280 cm^{-1} Raman band is parallel to the electric field, while the molecular vibration associated with the 360 cm^{-1} Raman band is perpendicular to the electric field, hence a positive and negative Stark tuning is expected, respectively. Figure S14 shows this positive and negative Stark tuning for the 280 and 360 cm^{-1} Raman bands, as they move in the opposite direction. These Raman spectroscopy measurements were conducted at the same applied potential and temperature, but at different locations at the Cu electrode surface. This indicates that there is an inhomogeneity in the experienced interfacial electric field per measurement spot, showing the necessity to perform these experiments at multiple measurements spots as has been done in this study.

The previously mentioned Gaussian fits resulted in values for the peak maxima for both the 280 and 360 cm^{-1} peaks. These values are plotted in S15, showing their dependency on applied potential and temperature. The 280 cm^{-1} peak position seems to change little with applied potential (-0.7 V and -0.95 V vs. RHE, blue and orange lines, respectively). The 360 cm^{-1} peak position, however, shifts more

drastically towards higher Raman shifts with higher applied potential. Both the 280 and 360 cm^{-1} peak position seem to shift towards lower Raman shift at the highest temperature measured, which might indicate a change in the interfacial potential at elevated temperatures.

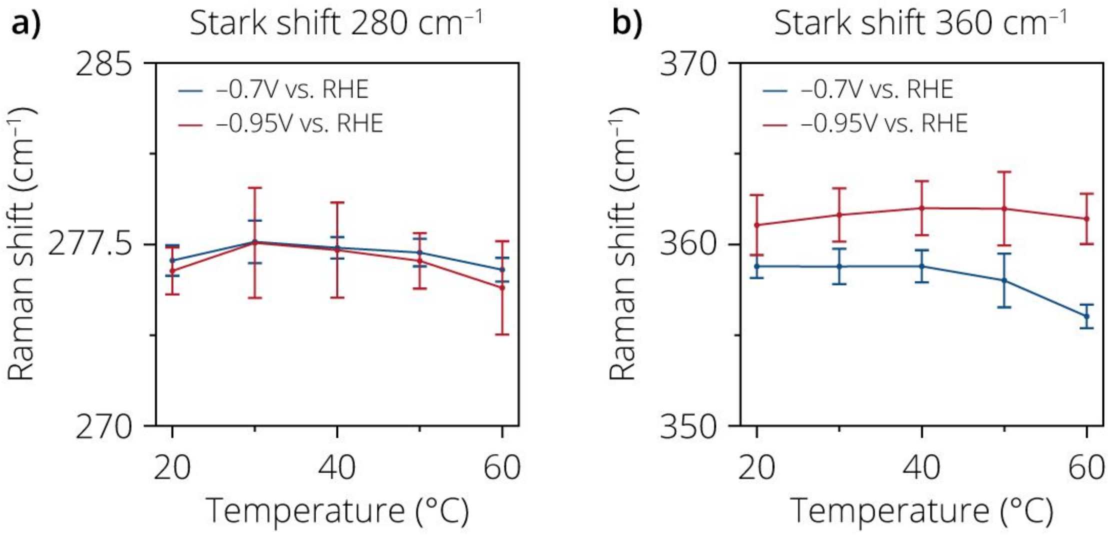

Figure S16: Temperature- and potential-induced Stark tuning of the 280 cm^{-1} (left) and 360 cm^{-1} (right) peak maxima. The blue lines show the measurements at -0.7V vs. RHE, the orange lines at -0.95V vs. RHE. The data points are the average of 5–10 spots measured at every potential and temperature and the error bar shows the standard deviation of the peak positions of these spots.

CO reduction experiments

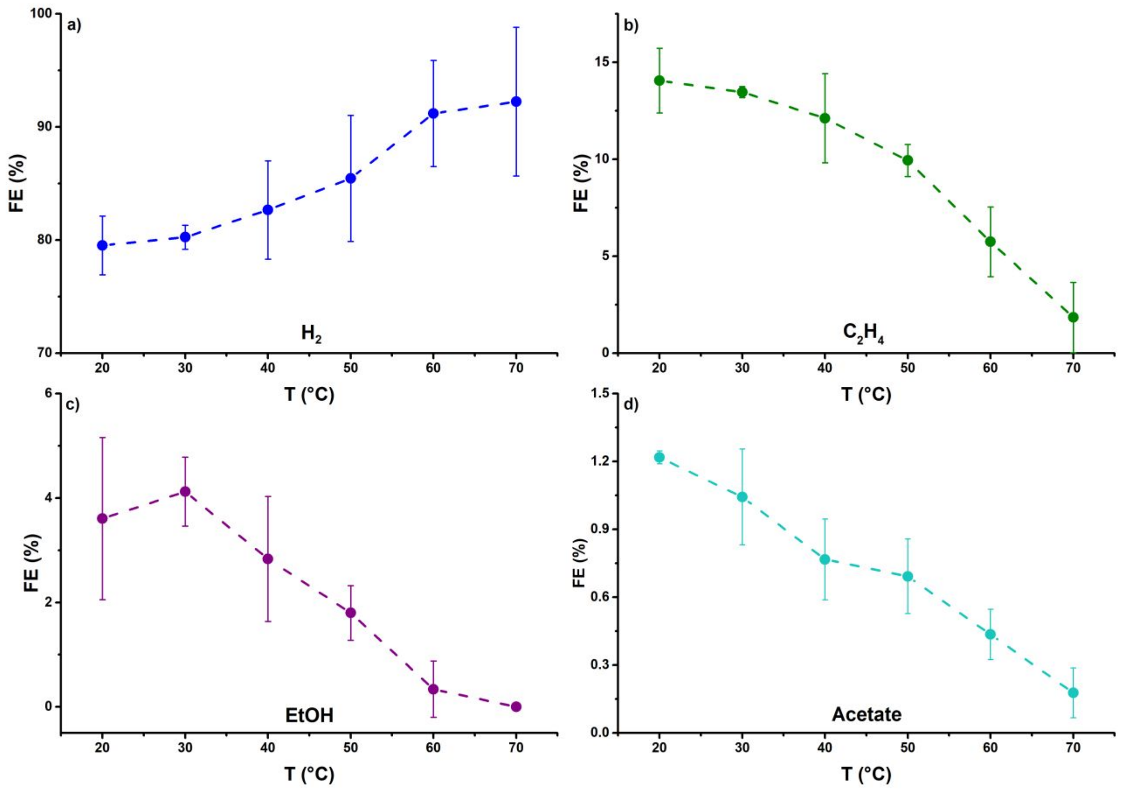

Figure S17: Faradaic efficiency of CO reduction at different temperatures in 0.1 M KOH + 0.2 M K_2SO_4 at -0.7 V vs. RHE for a) hydrogen b) ethylene c) ethanol, and d) acetate. Dotted lines are a guide to the eye and error bars are determined from at least 3 measurements.

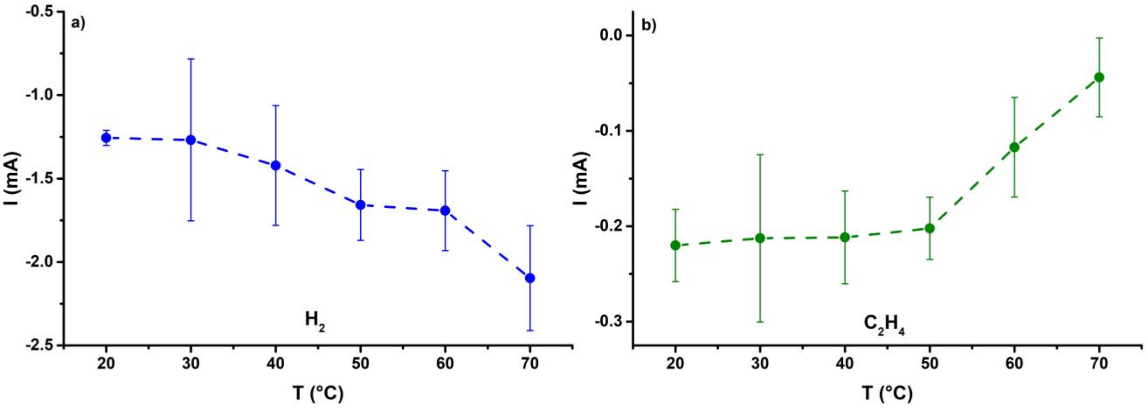

Figure S18: Currents for CO reduction at different temperatures in 0.1 M KOH + 0.2 M K_2SO_4 at -0.7 V vs. RHE for a) hydrogen and b) ethylene. Dotted lines are a guide to the eye and error bars are determined from at least 3 measurements.

Activation Energy

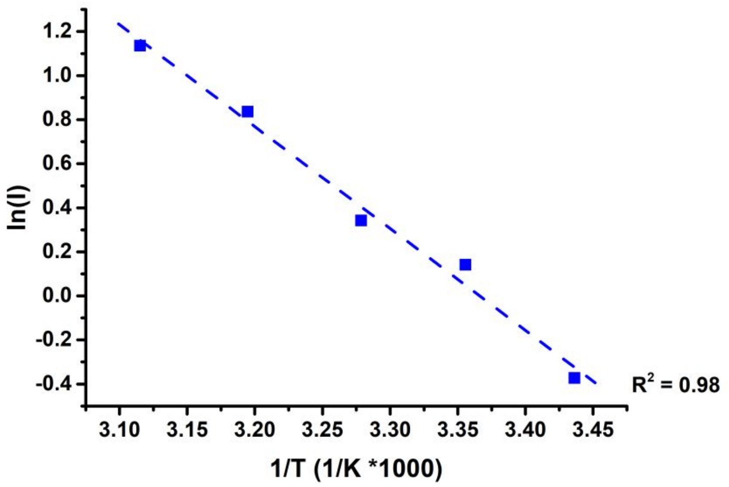

Figure S19: Arrhenius plot of H_2 production during CO2RR at -1.1 V vs. RHE in -0.1 M $KHCO_3$. The striped line gives the trendline with the corresponding R^2 value to illustrate the linearity of the trendline.

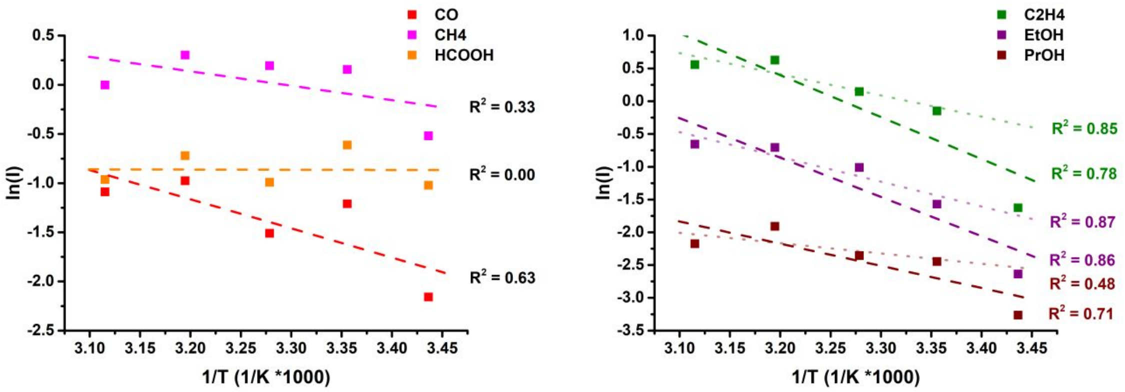

Figure S20: Arrhenius plots of a) CO, methane, formic acid production and b) ethylene, ethanol and 1-propanol during CO2RR at -1.1 V vs. RHE in -0.1M $KHCO_3$. The striped lines gives the trendline with the corresponding R^2 value to illustrate the linearity of the trendline. The light doted lines for the C2+ products are the trendlines without the point at 18 °C. It can be seen that the slope changes significantly.

Supporting discussion: We have shown previously that on a gold electrode, the (apparent) E_a for the CO2RR towards CO is higher than for the HER, which results in an increase in CO selectivity with increasing temperature. [4] However, Zong et al. [5] showed that on copper, the HER seems to have the highest apparent activation energy. We have tried to determine the apparent activation energy from our dataset as well, however this does not give straightforward results (Figure S19 and S20). Ethylene and ethanol give semi-linear trends up to 48 °C. If the data point at 18 °C is not considered, they exhibit E_a of 27 ± 7 and 31 ± 8 kJ/mol, respectively, comparable to the results by Zong et al.. However, including the 18 °C data point, the E_a increases to 53 ± 16 and 50 ± 12 kJ/mol, respectively, showing that our data set is not sufficient to determine a reliable E_a. Unfortunately, the other products show non-linear trends. Only for HER we are able to determine a relatively accurate value (Figure S19). Interestingly, the E_a for H_2 determined from our experiments is significantly lower than the value determined by Zong et al. (38 ± 2 kJ/mol vs. ~ 60 kJ/mol, respectively).

Pb UPD

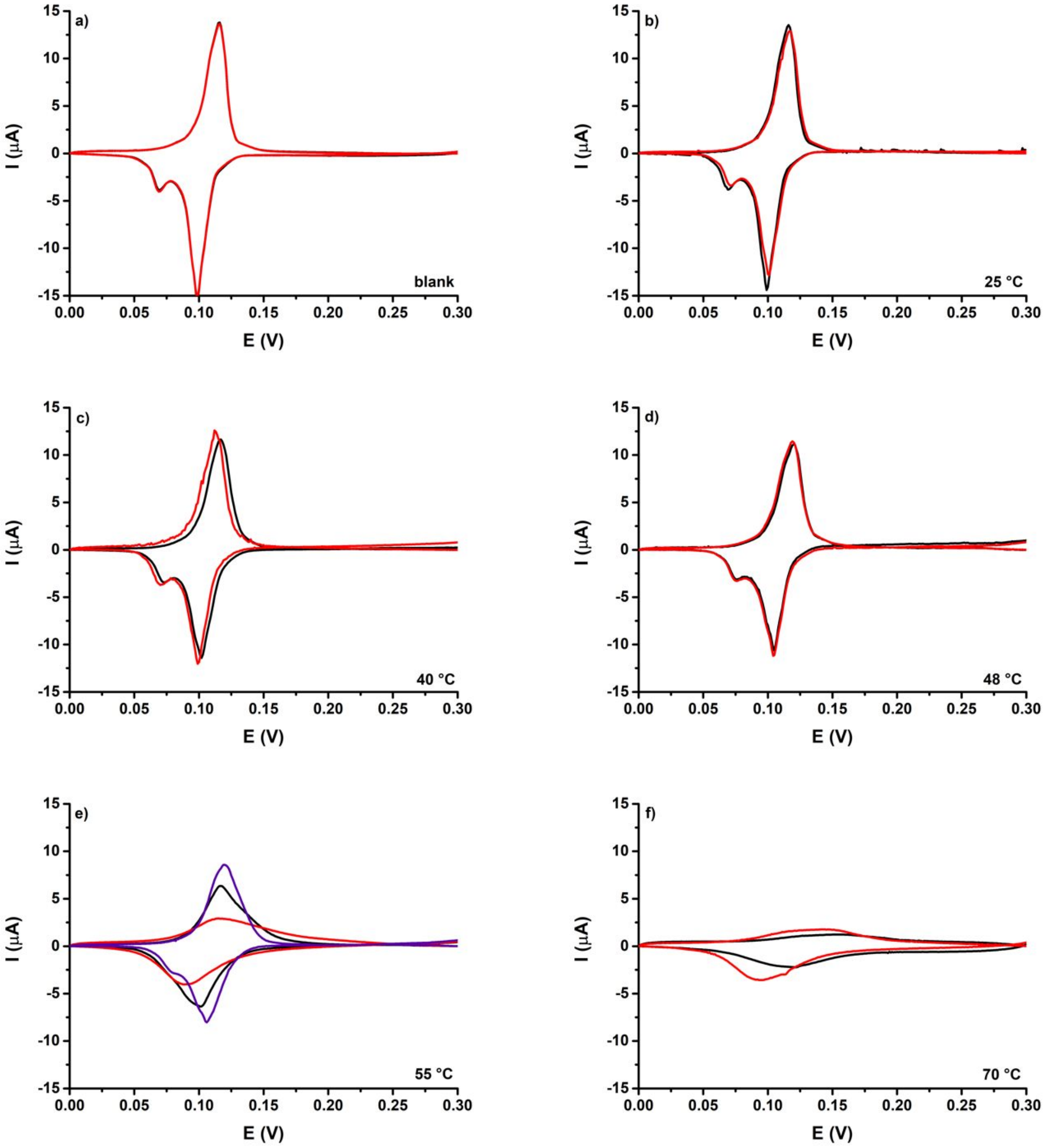

Figure S21: Pb UPD CVs from 0.3 to 0.0 V vs. RHE at 5 mV/s in 0.1 M $NaClO_4$ + 1mM NaCl + 2 mM $PbClO_4$. A) blank CV after only polishing before CO2RR and after CO2RR for 20 min at -1.1V vs. RHE in 0.1M $KHCO_3$ at b) 25 °C c) 40 °C d) 48 °C e) 55°C f) 70 °C. The red, black and purple lines are separate experiments to illustrate reproducibility.

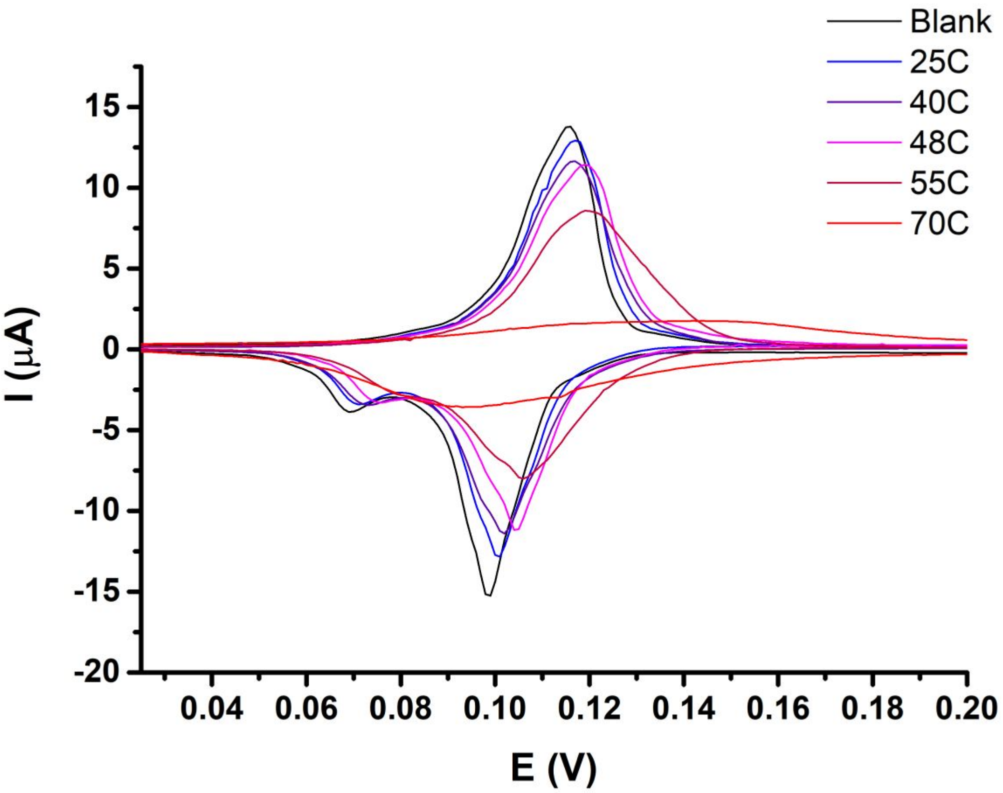

Figure S22: Pb UPD CVs from 0.3 to 0.0 V vs. RHE at 5 mV/s in 0.1 M $NaClO_4$ + 1mM NaCl + 2 mM $PbClO_4$. The copper sample was first used for CO2RR for 20 min at -1.1V vs. RHE in 0.1 M $KHCO_3$ at different temperatures, while the blank is before CO2RR was performed.

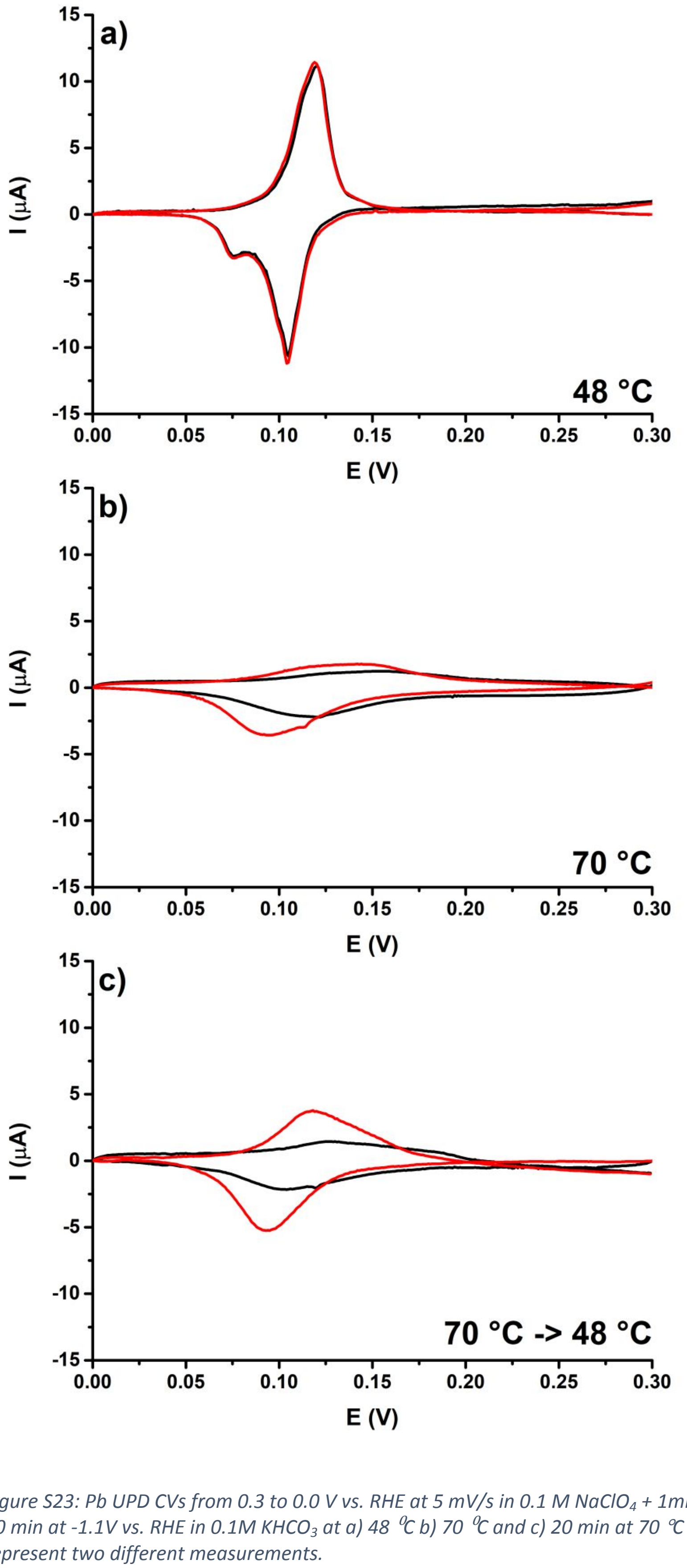

Figure S23: Pb UPD CVs from 0.3 to 0.0 V vs. RHE at 5 mV/s in 0.1 M $NaClO_4$ + 1mM NaCl + 2 mM $PbClO_4$. a) after CO2RR for 20 min at -1.1V vs. RHE in 0.1M $KHCO_3$ at a) 48 ⁰C b) 70 ⁰C and c) 20 min at 70 ℃ and 30 min at 48 ℃. The red and black line represent two different measurements.

SEM-EDX measurements

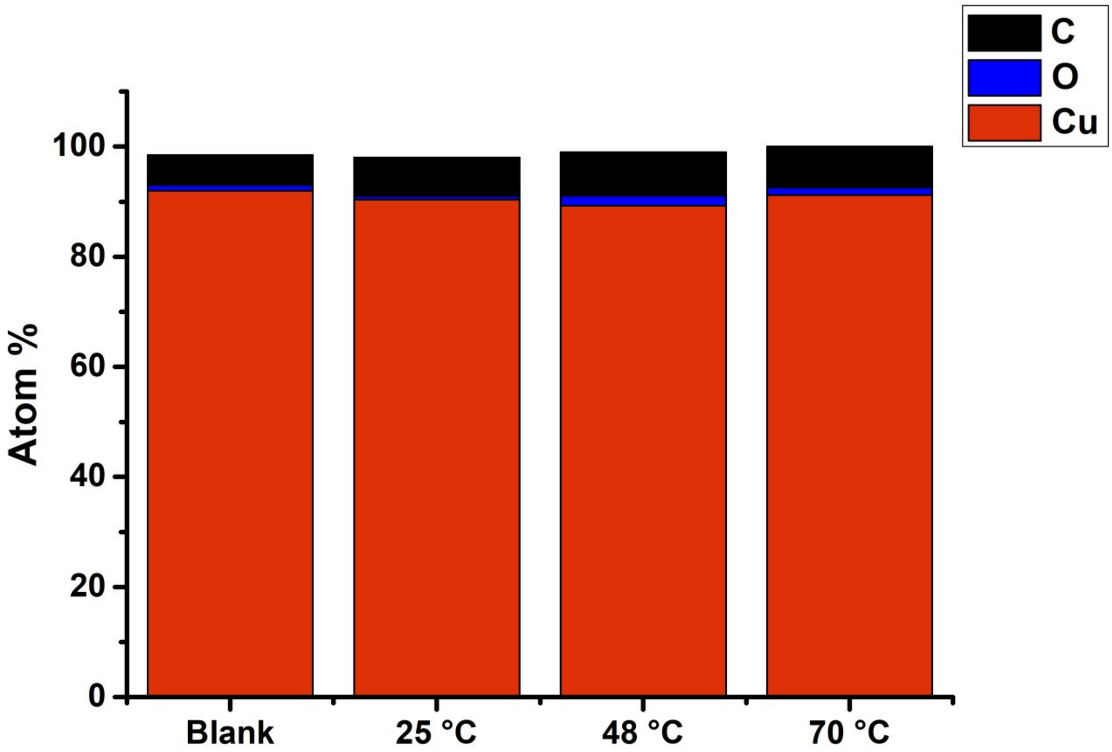

Figure S24: Elemental composition measured using SEM-EDX of the copper surface after CO2RR at -1.1V at different temperatures and the blank before CO2RR

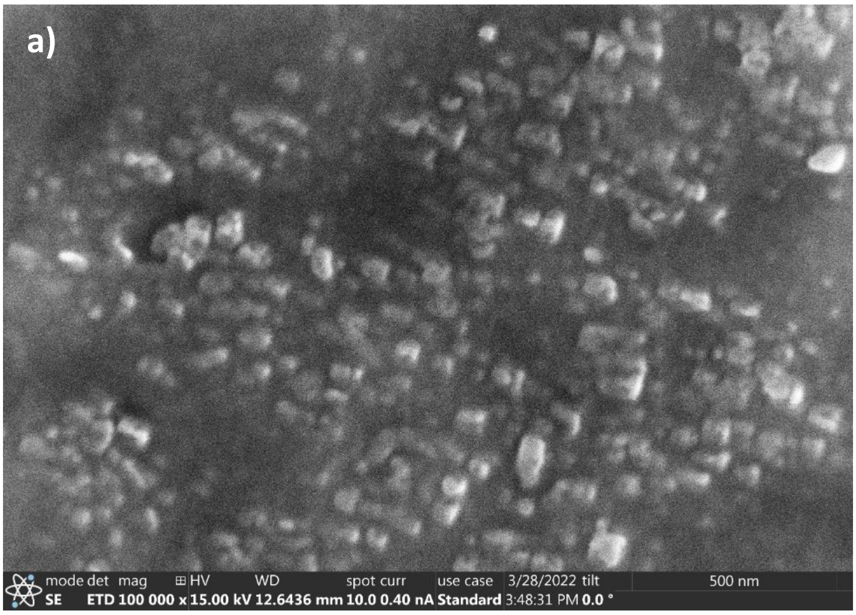

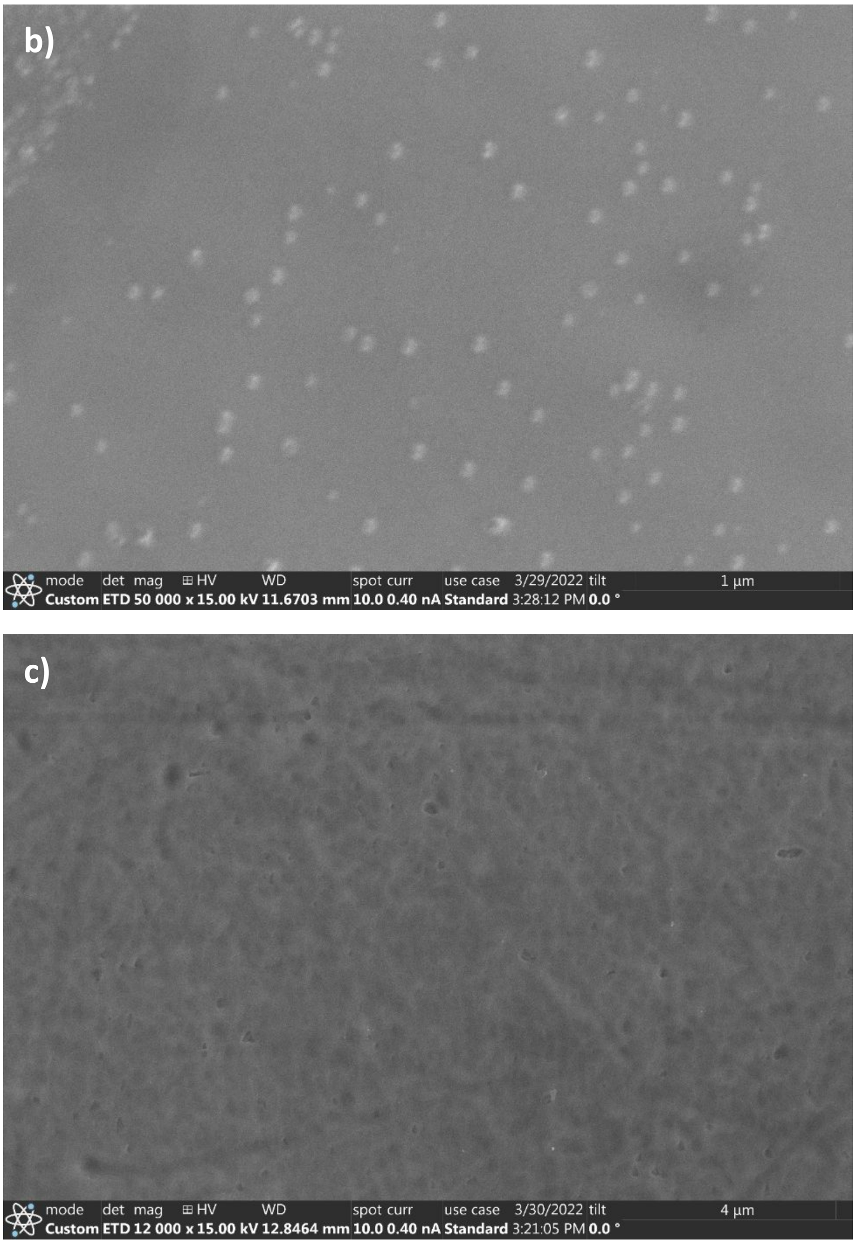

Figure S25: SEM micrographs after CO2RR at -1.1V vs. RHE at a) 25 ℃ b) 48 ℃ c) 70 ℃

Double layer capacitance measurements

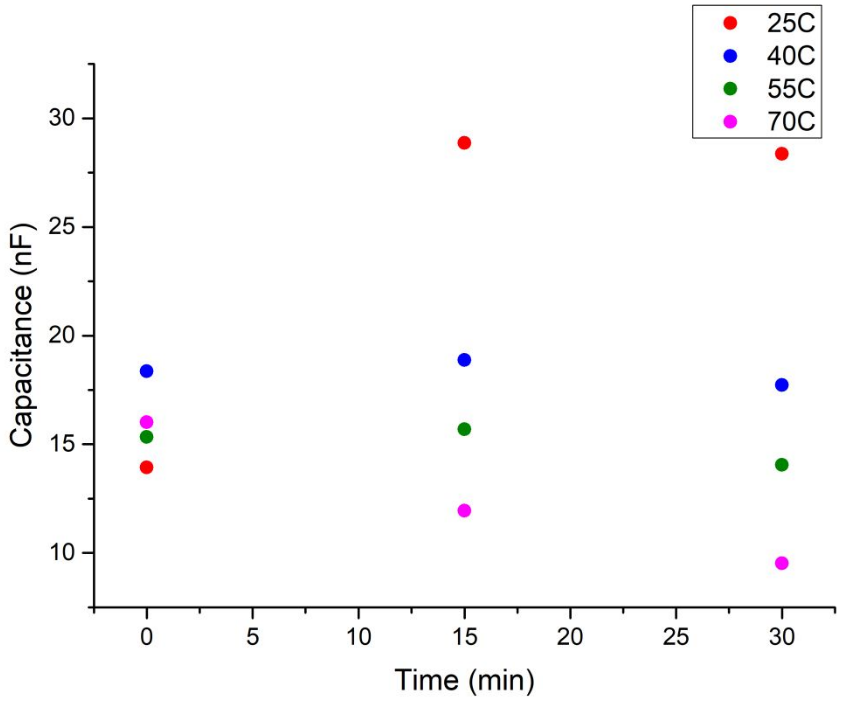

Figure S26: Capacitance measured by double layer capacitance studies from CVs in the range -0.2 to 0.3 V vs. RHE at scan rates from 200 to 1400 mV/s before and after 15 and 30 min of CO2RR at different temperatures. The capacitance was determined at 0.0 V vs. RHE.

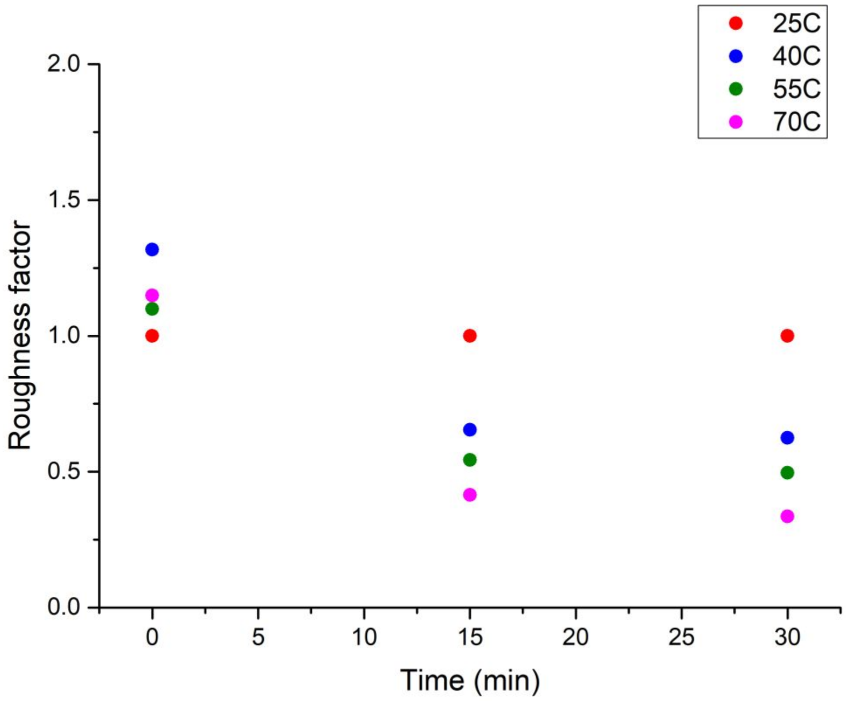

Figure S27: Roughness factor determined from the data from Figure S23 by setting the capacitance at 25 °C as 1 at every time interval and normalize the other values.

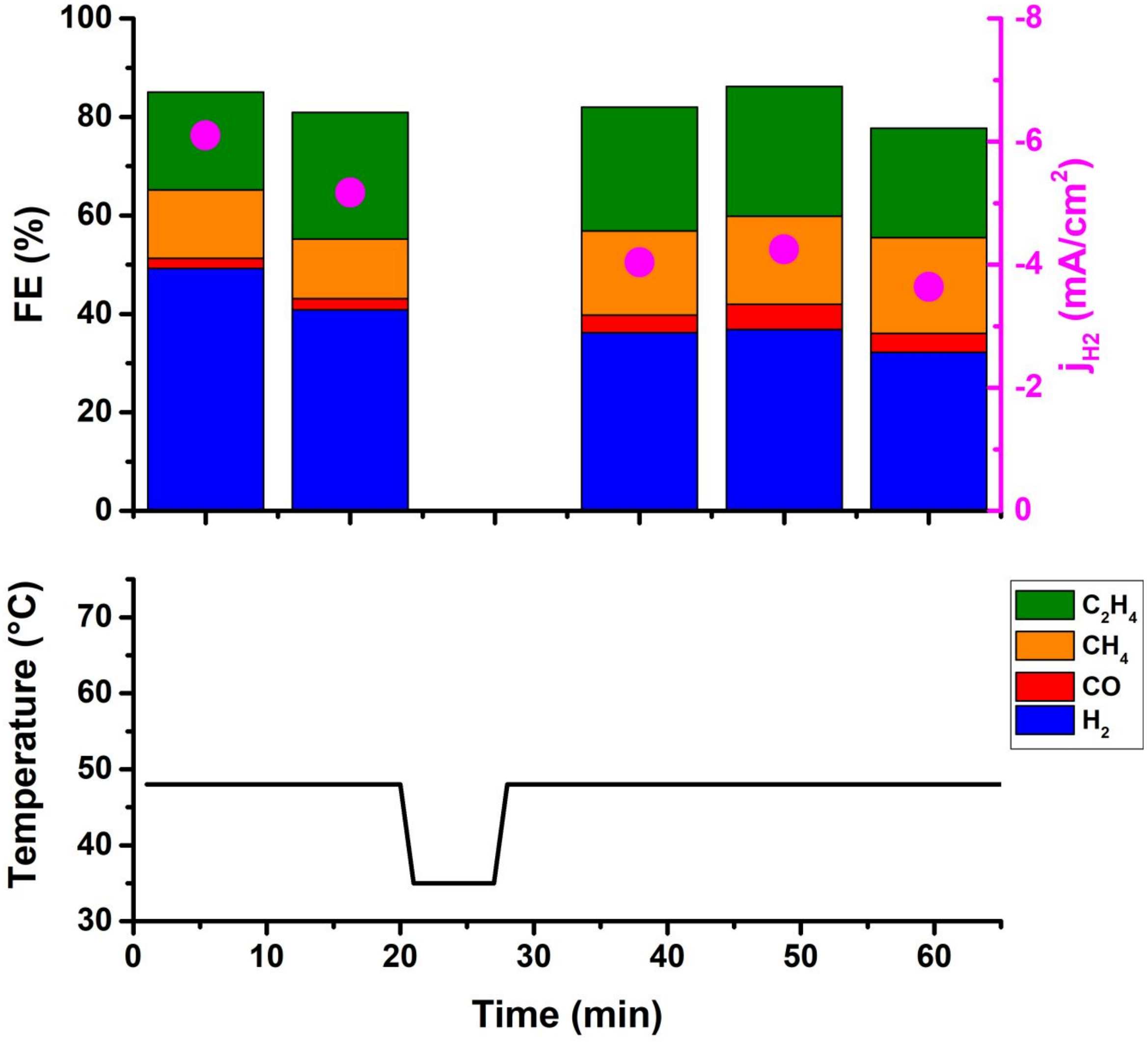

Figure S28: Reversibility experiments, control experiment to Figure 6. The upper panel shows the Faradaic Efficiency of the gaseous products of CO2RR at -1.1V vs. RHE after 5 and 19 min at 48 °C, and after cooling down at 5, 19 and 32 min at 48 °C. The magenta dots show the patial current density towards H_2. The lower panel shows the temperature profile.

References

(1) Caccia, M.; Ebolese, A.; Maspero, M.; Santoro, R.; Locatelli, M.; Pieracci, M.; Tintori, C. Background Removal Procedure Based on the SNIP Algorithm for γ–Ray Spectroscopy with the CAEN Educational Kit. *Educational Note* **2013**, *2*, ED3163.

(2) Chernyshova, I. V; Somasundaran, P.; Ponnurangam, S. On the Origin of the Elusive First Intermediate of CO_2 Electroreduction. *Proceedings of the National Academy of Sciences* **2018**, *115* (40), E9261–E9270.

(3) Chang, X.; Xiong, H.; Xu, Y.; Zhao, Y.; Lu, Q.; Xu, B. Determining Intrinsic Stark Tuning Rates of Adsorbed CO on Copper Surfaces. *Catal Sci Technol* **2021**, *11* (20), 6825–6831.

(4) Vos, R. E.; Koper, M. T. M. The Effect of Temperature on the Cation-Promoted Electrochemical CO_2 Reduction on Gold. *ChemElectroChem* **2022**, *9* (13), e20220023. https://doi.org/10.1002/celc.202200239.

(5) Zong, Y.; Chakthranont, P.; Suntivich, J. Temperature Effect of CO_2 Reduction Electrocatalysis on Copper: Potential Dependency of Activation Energy. *Journal of Electrochemical Energy Conversion and Storage* **2020**, *17* (4), 1–7. https://doi.org/10.1115/1.4046552.
